# The barley stripe mosaic virus expression system reveals the wheat C2H2 zinc finger protein TaZFP1B as a key regulator of drought tolerance

**DOI:** 10.1186/s12870-020-02355-x

**Published:** 2020-04-07

**Authors:** Arnaud Cheuk, Francois Ouellet, Mario Houde

**Affiliations:** Département des Sciences biologiques, Université du Québec à Montréal, C.P. 8888, Succ. Centre-ville, Montréal, Québec H3C 3P8 Canada

**Keywords:** Barley stripe mosaic virus, C2H2 zinc finger proteins, Drought, Functional characterization, Gene overexpression, Plant transformation, RNA-Seq, siRNA, Transcriptome, *Triticum aestivum*

## Abstract

**Background:**

Drought stress is one of the major factors limiting wheat production globally. Improving drought tolerance is important for agriculture sustainability. Although various morphological, physiological and biochemical responses associated with drought tolerance have been documented, the molecular mechanisms and regulatory genes that are needed to improve drought tolerance in crops require further investigation. We have used a novel 4-component version (for overexpression) and a 3-component version (for underexpression) of a barley stripe mosaic virus-based (BSMV) system for functional characterization of the C2H2-type zinc finger protein TaZFP1B in wheat. These expression systems avoid the need to produce transgenic plant lines and greatly speed up functional gene characterization.

**Results:**

We show that overexpression of *TaZFP1B* stimulates plant growth and up-regulates different oxidative stress-responsive genes under well-watered conditions. Plants that overexpress *TaZFP1B* are more drought tolerant at critical periods of the plant’s life cycle. Furthermore, RNA-Seq analysis revealed that plants overexpressing *TaZFP1B* reprogram their transcriptome, resulting in physiological and physical modifications that help wheat to grow and survive under drought stress. In contrast, plants transformed to underexpress *TaZFP1B* are significantly less tolerant to drought and growth is negatively affected.

**Conclusions:**

This study clearly shows that the two versions of the BSMV system can be used for fast and efficient functional characterization of genes in crops. The extent of transcriptome reprogramming in plants that overexpress *TaZFP1B* indicates that the encoded transcription factor is a key regulator of drought tolerance in wheat.

## Background

Bread wheat (*Triticum aestivum* L.) is one of the most important crops worldwide and global demand is increasing. It was estimated that cereal production needs to increase by at least 50% between 2005 and 2050 [1, 2]. However, achieving this goal is uncertain due to limited land resources and the impact of various abiotic and biotic stresses. Drought stress is one of the major environmental stresses limiting crop productivity worldwide [3], and the frequency of drought spells is expected to increase with global climate change [4]. In order to improve crop yield, we must increase our understanding of the genetic and molecular mechanisms underlying the responses and tolerance mechanisms to various abiotic stresses in crops.

Most genomic studies have focused on plant models or on crops with diploid genomes (e.g. *Arabidopsis thaliana* and *Oryza sativa*) [5, 6]. These studies have provided valuable insights into different biological processes associated with various abiotic stresses in plants. While many conserved pathways are shared between models and crops, divergent functions sometimes arise between homologous genes during the course of evolution [7]. This limits direct translation of functional characterization results from model species to crops, and suggests that the identification of functional gene orthologues in crop plants such as wheat requires species-specific studies. Wheat is a hexaploid organism that originated from hybridization events between ancestral genomes. These events provided genetic diversity and plasticity [8] which is key to the success of this crop under different ecological conditions [9]. The diverse gene pools of cultivated or ancestral wheat provide a great opportunity to identify stress-associated genes and improve our knowledge of gene networks that may contribute to increase wheat performance under diverse abiotic stress conditions.

Plants, as sessile organisms, need to evolve different strategies to cope with and adapt to environmental changes. Exposure to abiotic stress induces physiological and metabolic responses that are mediated through complex signal transduction networks involving a great number of molecules and stress-responsive genes [10–13]. Drought stress is initiated by water deficit in soil, resulting in osmotic stress. Moreover, inhibition of CO_2_ fixation during drought leads to disturbances in the electron transport chain and photosystem activities in chloroplasts, resulting in increased ROS production and accumulation [14], which could be harmful to plants. In the course of evolution, plants have adapted dynamic responses at the morphological, physiological, and biochemical levels, allowing them to survive under rapidly changing environmental conditions. Adaptive responses associated with tolerance traits include cuticular wax biosynthesis on leaf surfaces, improved osmotic adjustment ability and increased cell wall elasticity to maintain tissue turgidity [15, 16] via the synthesis and accumulation of xyloglucan endotransglucosylase/hydrolase (XTH), cellulose synthase, pectin esterase, expansin, soluble carbohydrates and osmoprotectants like proline and glycine betaine [16, 17]. The manifestation of these morphological or physiological responses involves processes starting from perception of stress to the expression of large numbers of genes that increase the chances of survival. Increasing evidence supports that transduction of the stress signal and plant responses are mediated by calcium and the activation of several Ca^2+^ sensors [18–20]. In *Arabidopsis*, a study showed that overexpression of the Calmodulin 1 (CaM1) gene positively regulates NADPH oxidase RbohF, leading to abscisic acid (ABA)-triggered ROS production and stomatal closure [21].

ABA is a key drought-induced signal modulating physiological responses that eventually lead to acclimation and stress tolerance. Accumulation of ABA in leaves directly regulates stomatal movement [22, 23] and reduces water loss, resulting in drought avoidance [24]. The relationship between ABA sensitivity and drought tolerance has been demonstrated by a study in the wild wheat *Aegilops tauschii* in which drought-tolerant accessions of *A. tauschii* show significantly higher ABA sensitivity than drought-sensitive lines, and tend to accumulate more stress-responsive gene transcripts [23]. This suggests that ABA sensitivity is regulated by the expression of different genes involved in ABA perception/signaling. In studies using transgenic lines, overexpression of genes involved in ABA signaling such as the aspartic protease *ASPG1*, the NADP-malic enzyme or an E3 ubiquitin ligase enhanced tolerance to drought stress [25–27]. In another study, the loss of function through antisense regulation or by mutation of the receptor-like kinase1 (RPK1) in *Arabidopsis* decreased ABA sensitivity, stomatal closure and the expression of several stress-inducible genes such as LEA-like proteins, peroxidase, RD26, DnaJ-like protein, cytochrome P450 and SOD [28]. Enzymes belonging to the SnRK2 protein kinase subfamily are major regulators of plant response to ABA by direct phosphorylation of various downstream targets including transcription factors, the NADPH oxidase RbohF, LEA-like proteins, DREB (Dehydration-Responsive Element-Binding protein), slow anion channel (SLAC)-associated genes and antioxidant enzyme genes [29–31]. These studies show that drought tolerance is governed by a complex gene regulatory network which is still poorly understood in wheat.

Transcriptional factors are the most important regulatory proteins that modulate the expression of specific sets of genes [32–35]. They have major roles in plant responses to abiotic stresses, where they convert stress-induced signals to cellular responses. Drought stress up-regulated gene expression is driven by transcription factors belonging to families of drought response element binding protein/C-repeat binding factors (DREB/CBF), basic leucine zipper (bZIP), myeloblastosis oncogene (MYB), NAM, ATAF1/2 and CUC (NAC), nuclear factor Y (NF-Y), zinc finger proteins (ZFP), and proteins containing the highly conserved amino acid sequence WRKYGQK (WRKY) [36–41] via ABA-dependent or ABA-independent pathways. Functional analysis of stress-inducible transcription factors should provide more information on the complex regulatory gene networks and their involvement in abiotic stresses. In soy, overexpression studies of DREB1-type transcription factors showed that they induce a number of target genes belonging to dehydrins/LEA families, chaperones, and enzymes involved in detoxification and synthesis of secondary metabolites [42]. Several WRKY transcription factors have been implicated as regulators of stress responses and senescence in different plants [43]. Overexpression of *GmWRKY27* reduces ROS levels and improves salt and drought tolerance in transgenic soy plants [44]. In transgenic rice plants, overexpression of *OsWRKY89* leads to growth retardation, increased wax deposition on leaf surfaces, and ultraviolet B tolerance [45]. Another rice transcription factor, OsMYB2, confers tolerance to multiple stresses such as salinity, cold and drought by stimulating the accumulation of soluble sugars and proline [46], while overexpression of *Arabidopsis MYB96* enhances drought tolerance via cuticular wax accumulation [47]. Similarly, the rice SERF1 transcription factor has been demonstrated to regulate the expression of different genes associated with salt tolerance including three Cys2/His2-type (C2H2) zinc finger proteins (*ZFP179, ZFP182, ZFP252)* (Schmidt et al., 2013). Overexpression of these C2H2 ZFP transcription factors in rice was shown to increase tolerance to salt and/or drought [48, 49]. Additional studies suggest that C2H2 ZFP transcription factors are involved in responses and tolerance to drought, cold, salt, high light and oxidative stresses in *Arabidopsis thaliana* [50–54] and rice [48, 55–57]. Genetic analysis revealed that ZAT10 and ZAT12, two widely studied members of the C2H2 ZFPs family in *Arabidopsis*, are required for the expression of genes encoding ROS-scavenging enzymes [52, 58, 59]. These results suggest that C2H2 ZFPs could play important roles in regulation of ROS signaling under abiotic stress. In wheat, at least 53 members of a C2H2 *TaZFP* subfamily (C12i) have been identified [60]. The latter study revealed that 37 *TaZFP* members are up-regulated by drought stress and by at least one other abiotic stress. However, the mechanisms by which this *TaZFP* subfamily coordinates stress responses in wheat is poorly understood. Among these 37 members, *TaZFP1B* (*TaZFP1* from the B genome) showed strong expression under all stresses studied (high light, flooding, drought, H_2_O_2_), and was previously associated with Al tolerance [61]. This indicates that this gene could govern expression of stress-inducible genes and may play a significant role in various abiotic stresses with an oxidative stress component in wheat. Another wheat *ZFP* gene named *TaZFP1* was recently shown to improve salt stress tolerance in tobacco [62]. However, this TaZFP1 has 8 C2H2 domains (compared to two C2H2 domains in TaZFP1B) and has no significant homology with TaZFP1B or any other member of the TaZFP subfamily that we previously identified [60].

Here, we focused on the functional characterization of the C2H2 zinc finger transcription factor TaZFP1B in response to drought stress in wheat using a novel four-component BSMV overexpression system and the well-characterized three-component BSMV system for gene down-regulation [63]. Our results show that TaZFP1B improves tolerance to drought stress by stimulating scavenging ROS systems and by up-regulating numerous genes which were shown to improve drought and ROS tolerance in transgenic studies using different plant species [64]. Evidence show that TaZFP1B is a key regulator of drought tolerance in wheat.

## Results

### *TaZFP1B* expression levels are positively associated with increased drought stress tolerance

**Fig. 1:**
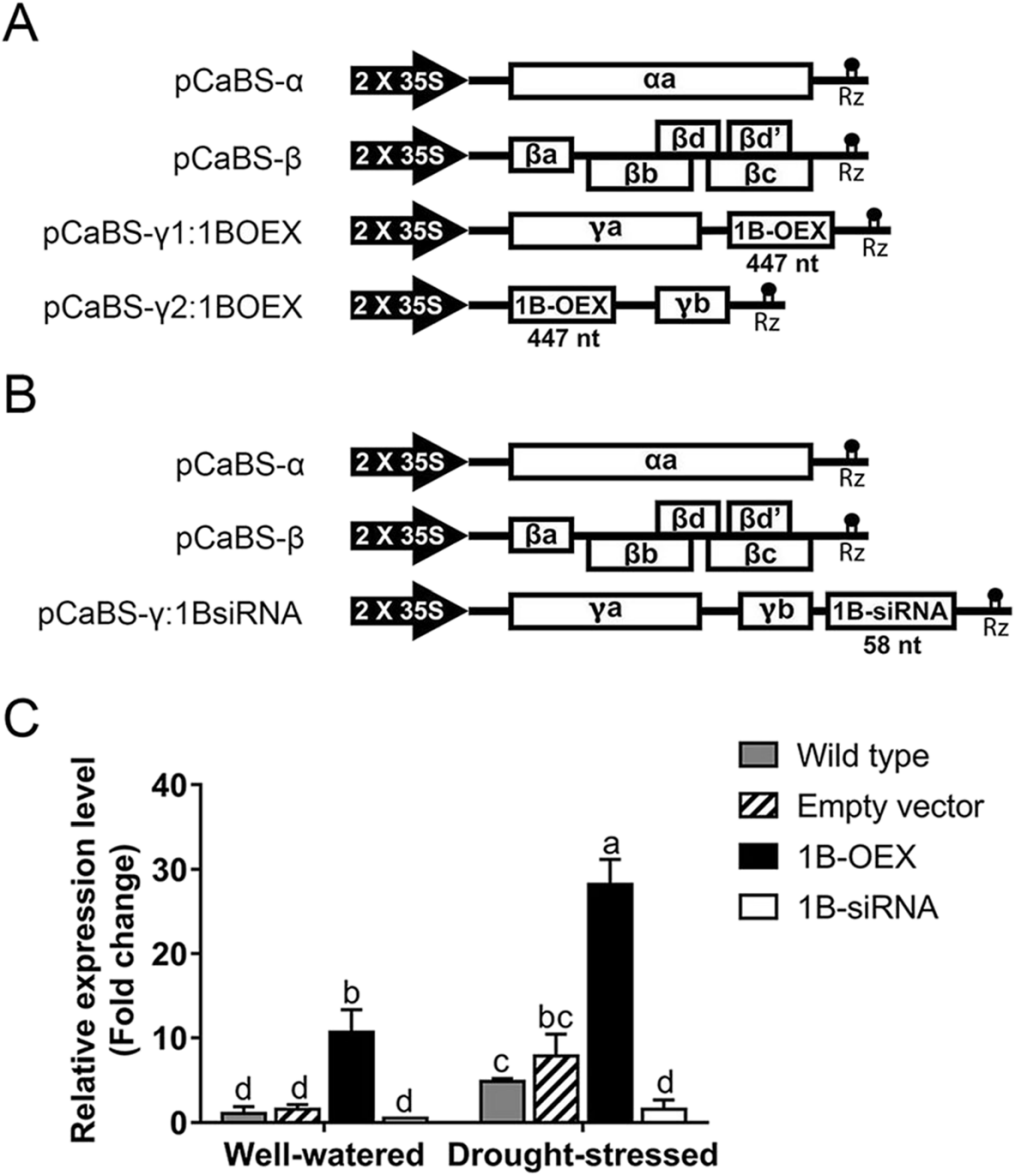
Schematic representations of the vectors used to modify *TaZFP1B* expression in wheat. **a** Vectors of the four-component BSMV system used for overexpression. **b** Vectors of the three-component BSMV system used for silencing. **c** Quantification of *TaZFP1B* transcripts in wheat. Wild-type, uninfected Atlas 66 plants; Empty-vector, Atlas 66 plants infected with the four basic (“empty”) plasmids: pCaBS-α, pCaBS-β, pCaBS-γ1:00 and pCaBS-γ2:00; 1B-OEX, Atlas 66 plants infected with the four plasmids described in (**a**); 1B-siRNA, Atlas 66 plants infected with the three plasmids described in (**b**). Values are means ±SD of four biological replicates. Different letters indicate statistically significant differences between samples (*P* < 0.05 by Tukey’s test)

**Fig. 2:**
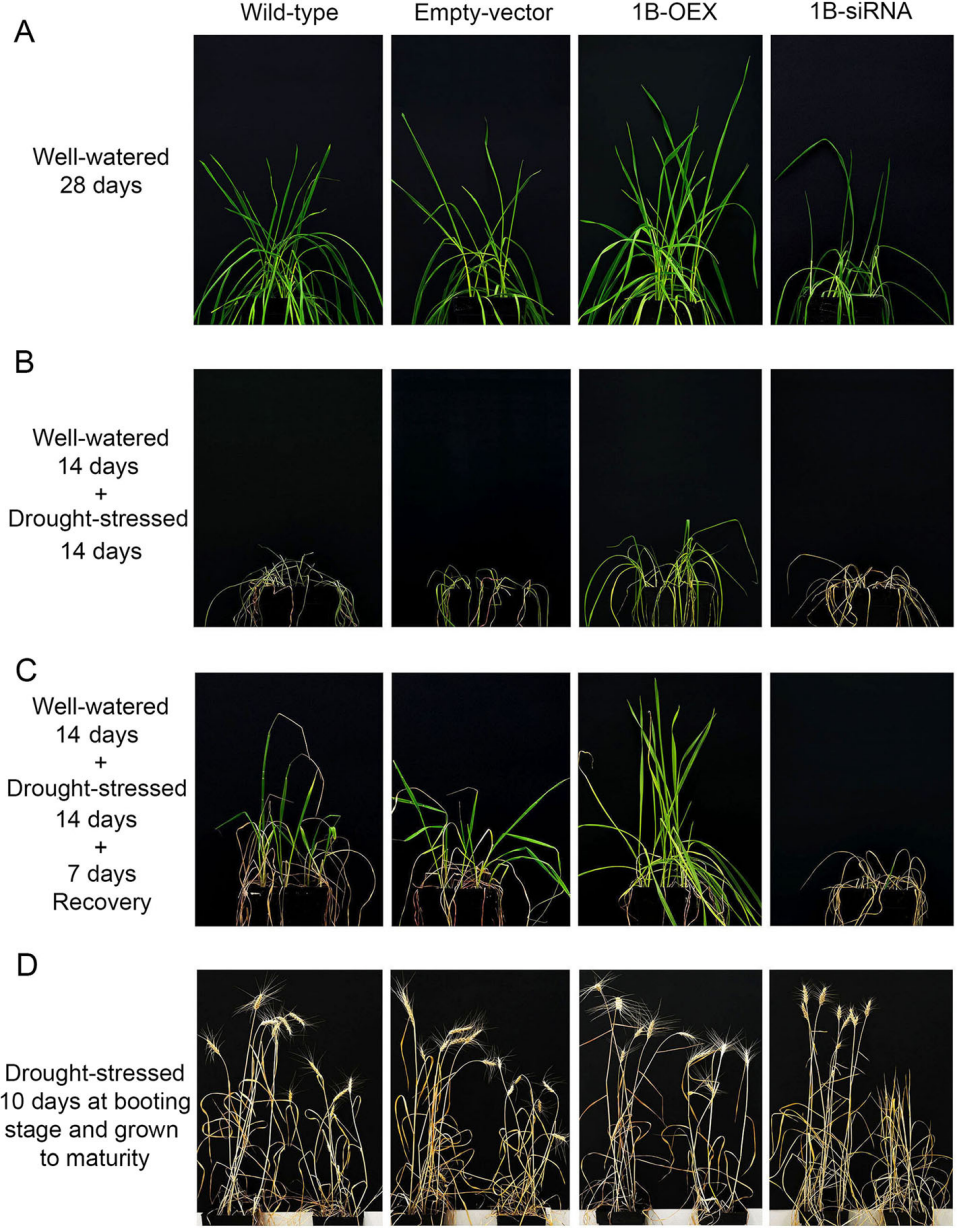
TaZFP1B enhances the tolerance of wheat plants to drought stress. The different types of wheat plants (see Fig. 1) were grown for 14 days then were either well-watered for an additional 7 days (**a**) or drought-stressed by withholding water for 14 days (**b**). For recovery, control plants and drought-stressed plants were watered every day for an additional 7 days (total of 35 days of growth) (**c**). Photograph of wild-type, empty vector, 1B-OEX and 1B-siRNA plants (cv. Dakosta) subjected to drought stress for 10 days at the booting stage and then grown to maturity (**d**)

To investigate the effects of TaZFP1B on drought stress tolerance, the three or four-component system of the barley stripe mosaic virus was used to generate wheat plants with lower or higher *TaZFP1B* expression levels, respectively (Fig. 1a and b). These transformants were named empty vector (no cDNA insert), 1B-OEX (*TaZFP1B* overexpression) and 1B-siRNA (*TaZFP1B* silencing). To confirm that the new BSMV virus-mediated overexpression (VOX) system can be used to modify *TaZFP1B* expression, this transcript was analyzed by qRT-PCR (Fig. 1c). As expected, the *TaZFP1B* transcript level in 1B-OEX plants was about 12-fold higher than the level observed in wild-type plants under well-watered conditions. Also as expected, *TaZFP1B* expression in 1B-siRNA plants is low in well-watered plants, confirming an efficient targeting of the *TaZFP1B* transcripts by the siRNA. The siRNA specificity was verified by analyzing the expression profiles of the closest *TaZFP* relatives (*TaZFP1A*, *1D*, *2B*, *2D*, *3B* and *3D*) (Additional file 1: Fig. S1). This analysis revealed that the 1B-siRNA also affected the expression of the homoeologous copy *TaZFP1A* (from the A genome) but did not target *TaZFP2* or *TaZFP3* transcripts [60]. Note that *TaZFP2A* and *TaZFP3A* have not been identified in wheat. Following a 7-day drought treatment, the *TaZFP1B* transcript level in wild-type plants was up-regulated by about 5-fold compared to well-watered plants. A similar result was observed in drought-stressed empty vector plants. On the other hand, the drought-induced up-regulation of *TaZFP1B* in 1B-OEX plants was about 6-fold higher than the up-regulation observed after drought stress in wild-type plants. We then sought to determine if an increased expression in *TaZFP1B* causes phenotypic changes (Fig. 2). Interestingly, overexpression of *TaZFP1B* improved plant growth under well-watered conditions (Fig. 2a). In 1B-OEX plants, the growth rate was more vigorous compared to the three other plants (wild-type, empty-vector and 1B-siRNA). On the other hand, silencing of *TaZFP1B* caused a slight loss of turgor. A 14-day drought stress significantly impaired the turgor of the four plant types, but the effect was less severe in 1B-OEX plants (Fig. 2b). Upon rewatering for 7 days, 1B-OEX plants recovered faster than the other plants (Fig. 2c). The fact that there is no visible phenotypic difference between the empty vector and wild-type plants indicates that the phenotypes under well-watered and drought conditions depend on *TaZFP1B* expression levels and not on the viral components used for infection. To verify the effect of drought on seed yield, water was withheld for 10 days after plants reached the booting stage (Fig. 2d). Our results show that drought stress significantly reduced the spike length, the total weight of grains per spike in all plants, the number of grains per spike and the average grain weight (weight of 10 grains). Interestingly, the total weight of grains per spike was higher in 1B-OEX plants while it was lower in 1B-siRNA plants compared to wild-type and empty vector plants (Table 1). Again, *TaZFP1* expression levels likely explain the results obtained since the data are similar between empty-vector and wild-type plants, which show similar levels of *TaZFP1B* expression.

**Table 1:** *TaZFP1B* transcript levels affect the wheat inflorescence parameters. Plants (cv. Dakosta) were grown and treated as described in Fig. 2

	Wild-type		Empty-vector		1B-OEX		1B-siRNA	
	Well-watered	Drought	Well-watered	Drought	Well-watered	Drought	Well-watered	Drought
Spike length (cm)	7.10 ± 1.00^a^	4.35 ± 0.58^c^	6.64 ± 0.63^a^	4.40 ± 0.65^c^	6.89 ± 0.74^a^	5.54 ± 0.58^b^	5.57 ± 0.44^b^	3.35 ± 0.48^d^
Total weight of grains per spike (g)	0.53 ± 0.17 ^b^	0.19 ± 0.04 ^d^	0.61 ± 0.11^ab^	0.17 ± 0.01 ^d^	0.71 ± 0.08^a^	0.30 ± 0.07^c^	0.41 ± 0.10^c^	0.08 ± 0.03 ^e^
Number of grains per spike	17.50 ± 5.68^ab^	9.90 ± 2.23^c^	19.17 ± 1.11^ab^	9.29 ± 1.11^c^	21.50 ± 4.50^a^	12.36 ± 3.11^bc^	16.14 ± 4.22^b^	7.14 ± 2.12^d^
Weight of 10 grains (g)	0.30 ± 0.03^a^	0.19 ± 0.02^c^	0.32 ± 0.04^a^	0.18 ± 0.02^c^	0.33 ± 0.02^a^	0.24 ± 0.02^b^	0.26 ± 0.03^b^	0.11 ± 0.01^d^

Values represent mean ± SD (*N* = 8). Different letters indicate significant differences between groups (*P* < 0.05)

**Fig. 3:**
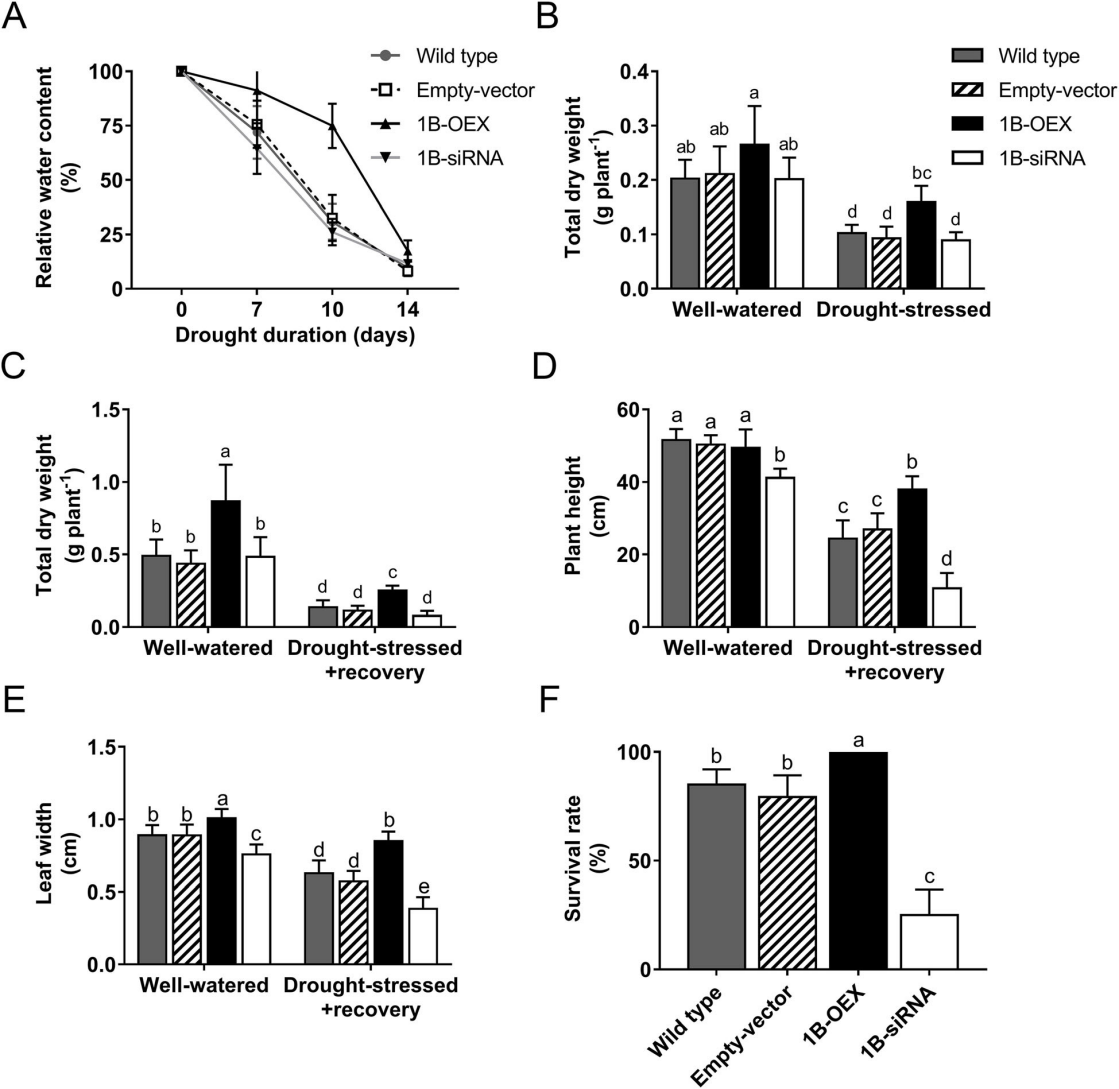
*TaZFP1B* overexpression improves the plants’ physiological parameters. Plants were grown and treated as described in Fig. 2. The following physiological parameters were measured: **a** relative water content; **b** total dry weight in well-watered and drought-stressed plants; **c** total dry weight in well watered plants and after drought-stress recovery; **d** plant height; **e** 2nd leaf width; and **f** survival rate after drought treatment and recovery. Values are means ±SD of four biological replicates. Different letters indicate statistically significant differences between samples (*P* < 0.05 by Tukey’s test)

The effect of drought stress on other growth parameters was investigated. The relative water content (RWC) is an important indicator of water status. Plants were drought-stressed for 14 days and the RWC was determined at different time points. Results in Fig. 3a show that the RWC decreases quickly in the wild-type, empty vector and 1B-siRNA plants compared to 1B-OEX plants. A drought stress of 10 days resulted in a significantly higher RWC in 1B-OEX plants compared to the other plants. Results in Fig. 3b show that dry weight accumulation was not significantly different between the four types of plants under well-watered conditions. However, exposure of plants to drought stress for 14 days resulted in a reduction in dry weight compared to the well-watered plants, albeit this reduction was significantly less in 1B-OEX plants. After 35 days of growth under well-watered conditions, the dry weight was higher 1B-OEX plants than in the other plants, and this was also the case after 14 days of drought stress and 7 days of recovery (Fig. 3c). Despite the superior performance of 1B-OEX plants, there was a significant reduction in dry weight in drought-stressed + recovery plants compared to 1B-OEX well-watered plants that continue to grow under optimal conditions (Fig. 3c). There was a significant reduction in plant height in all plants after drought stress and recovery compared to well-watered plants. However, the plant height of the 1B-siRNA plants was lower compared to the three other types of plants under well-watered conditions or after drought-stress and recovery (Fig. 3d). After drought stress and recovery, 1B-OEX plants were the tallest, while the 1B-siRNA plants were the shortest. Under well-watered conditions, the 1B-OEX plants also developed wider leaves while the opposite effect occurred in the 1B-siRNA plants (Fig. 3e). After drought stress and recovery, the leaf width was reduced in all plants compared to well-watered plants. However, leaves of the 1B-OEX plants were the widest while they were the narrowest in the 1B-siRNA plants. After drought stress and recovery, all the 1B-OEX plants survived while the survival rate for the 1B-siRNA was only at 25% compared to 80–85% in wild-type and empty vector plants (Fig. 3f). Moreover, chlorophyll fluorescence imaging showed that a 10-day drought stress decreases chlorophyll content in the leaves of wild-type and empty vector plants, and even more sharply in 1B-siRNA plants (Additional file 2: Fig. S2). In contrast, chlorophyll fluorescence remains high in 1B-OEX plants even after 14 days of drought. These results suggest that TaZFP1B participates in chlorophyll stability under stress.

### TaZFP1B improves tolerance to drought-induced oxidative stress

**Fig. 4:**
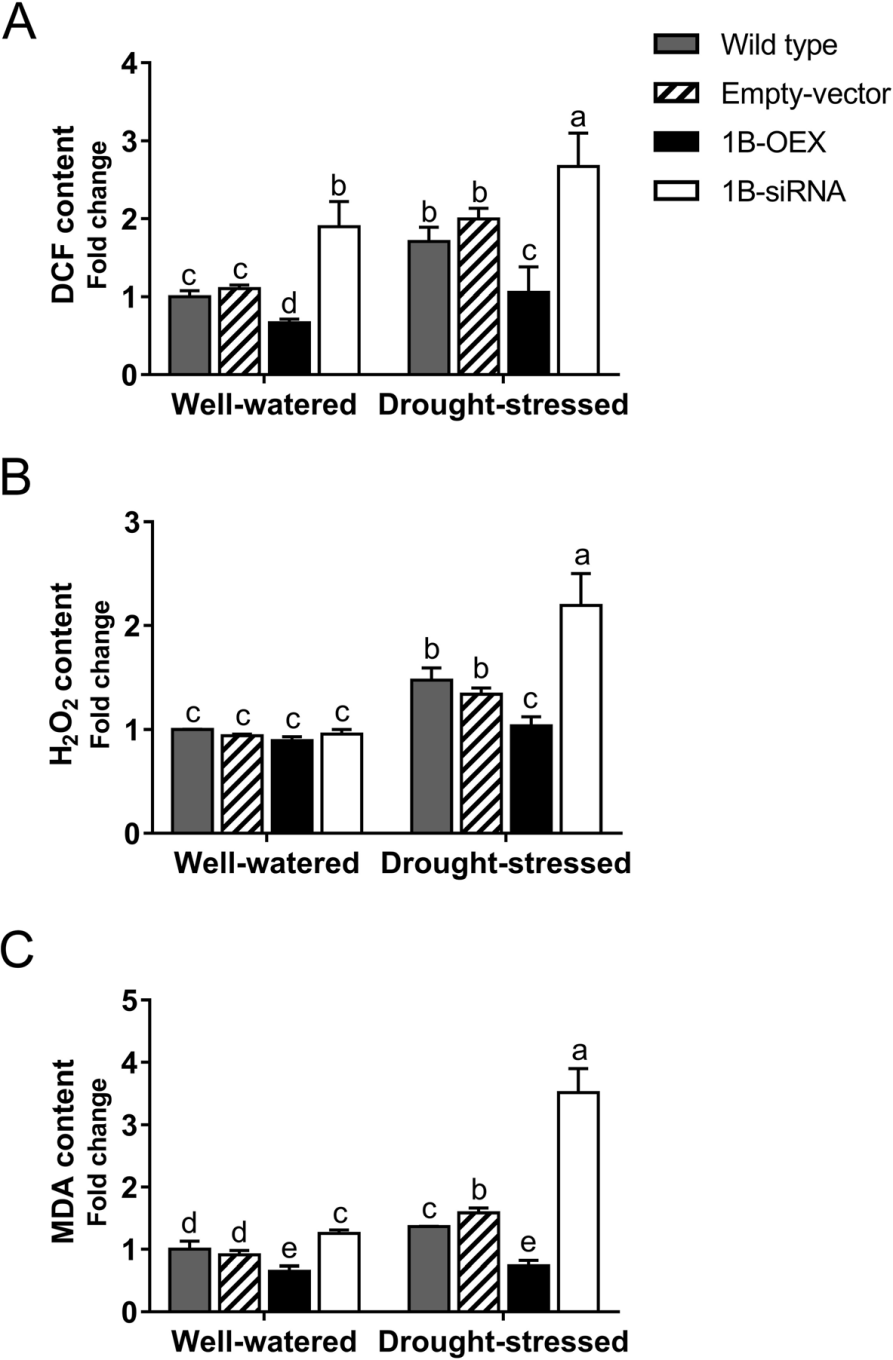
TaZFP1B reduces ROS accumulation under drought stress. Plants were grown and treated as described in Fig. 2 except that drought stress was applied for 7 days. Soluble extracts were prepared and contents in DCF (**a**), H_2_O_2_ (**b**) and malondialdehyde (**c**) were determined, as well as the protein content and the results are expressed as fold-change relative to well watered wild-type plants. Values are means ±SD of four biological replicates. Different letters indicate statistically significant differences between samples (*P* < 0.05 by Tukey’s test)

The effect of TaZFP1B on ROS accumulation was investigated by analyzing the accumulation of 2′,7′-dichlorofluorescein (DCF) (Fig. 4a). Well-watered wild-type and empty vector plants show similar levels of DCF, while lower and higher levels of DCF were observed in 1B-OEX and 1B-siRNA plants, respectively. A 7-day drought stress led to a significant accumulation of DCF in wild-type and empty vector plants compared to well-watered plants. The DCF level was much higher in 1B-siRNA plants, while in contrast it was significantly lower in 1B-OEX plants.

Since H_2_O_2_ is the most stable of the major ROS species produced under drought stress [65, 66], its production in leaf tissues was also examined (Fig. 4b). There are no significant changes in H_2_O_2_ content between the different well-watered plants. After 7 days of drought stress, there was a significant increase in H_2_O_2_ in wild-type and empty vector plants, and the highest level of H_2_O_2_ was observed in the 1B-siRNA plants. In contrast, there was no significant increase in H_2_O_2_ in the 1B-OEX plants.

The accumulation of various free radicals results in lipid peroxidation, which itself causes the formation of by-products such as malondialdehyde (MDA) [67]. Oxidative damage to lipids was estimated by measuring the MDA content in leaf tissues. As shown in Fig. 4c, well-watered 1B-OEX plants show reduced MDA accumulation and 1B-siRNA plants show increased accumulation. After drought stress, higher levels of MDA were observed in wild-type and empty vector plants, and even more so in the 1B-siRNA plants. In contrast, overexpression of *TaZFP1B* prevented drought-induced MDA accumulation.

### Gene expression and activities of ROS scavenging systems

**Fig. 5:**
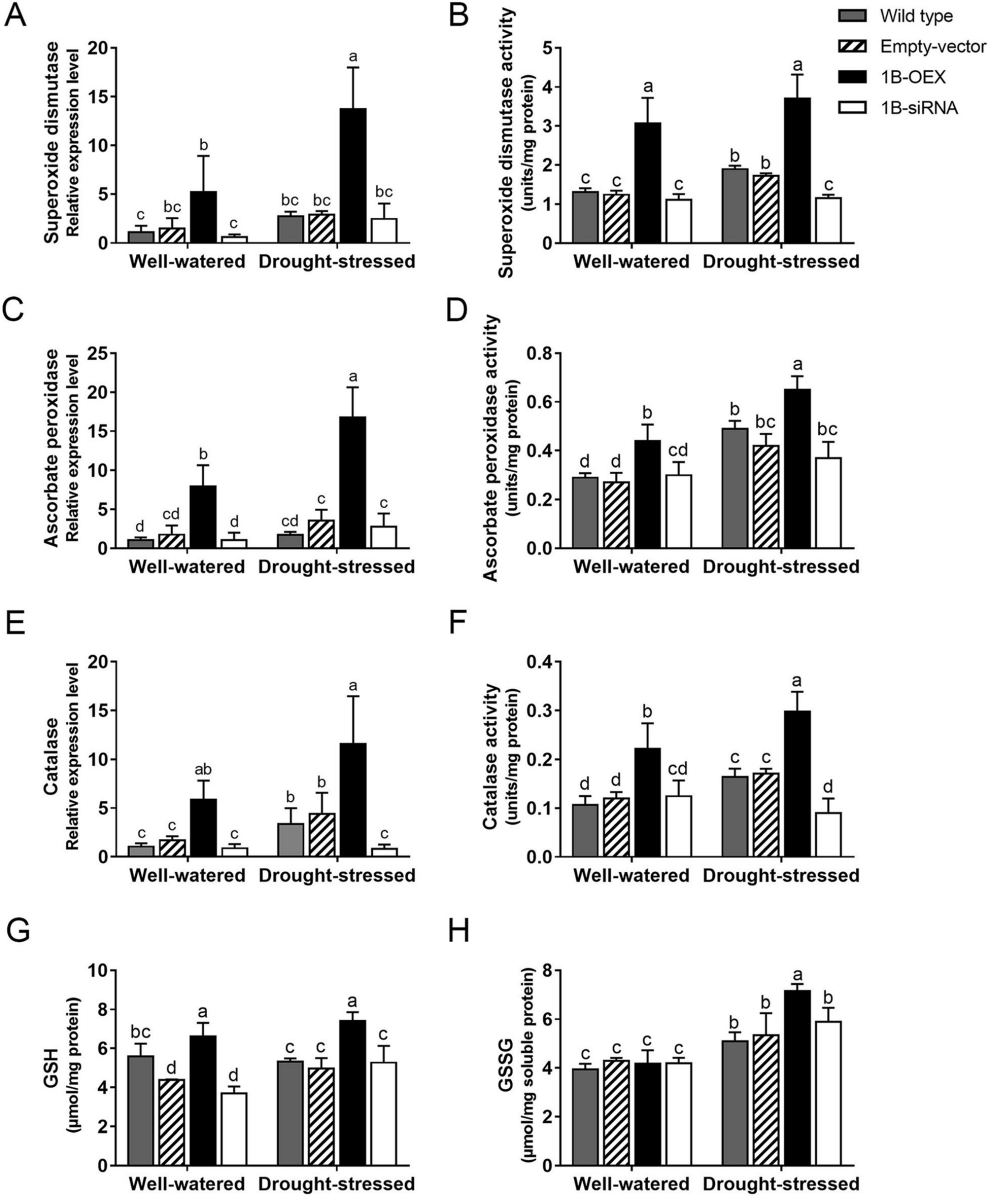
TaZFP1B enhances the gene expressions and activities of ROS scavenging systems. Plants were grown and treated as described in Fig. 2 except that drought stress was applied for 7 days. Expression levels of *SOD* (**a**), *APX* (**c**) and *CAT* (**e**) were determined by qPCR and and the results are expressed as fold-change relative to well watered wild-type plants. The enzyme activity corresponding to these genes was also assayed (**b**, **d** and **f**). The contents in reduced and oxidized glutathione (GSH and GSSG) were also determined (**g** and **h**). Values are means ±SD of four biological replicates. Different letters indicate statistically significant differences between samples (*P* < 0.05 by Tukey’s test)

The presence of antioxidant enzymes and compounds is needed to maintain cellular ROS homeostasis under stress conditions [11]. Major ROS-scavenging enzymes of plants include superoxide dismutase (SOD), ascorbate peroxidase (APX) and catalase (CAT). To investigate whether TaZFP1B is required for ROS scavenging, genes encoding SOD (TRIAE_CS42_2BL_TGACv1_131439_AA0427700), APX (TRIAE_CS42_U_TGACv1_642188_AA2112960.5) and CAT (TRIAE_CS42_7DL_TGACv1_602975_AA1973160.1), which showed increased expression in RNA-Seq data, were selected for analysis by qRT-PCR (Fig. 5a, c and e respectively). Enzymatic activities of SOD, APX and CAT were also determined (Fig. 5b, d and f respectively). In well-watered conditions, a significant up-regulation of *SOD* expression and total SOD activity was observed in 1B-OEX plants while there is no significant difference in the three other types of plants (Fig. 5a and b). A 7-day drought stress up-regulated *SOD* RNA expression and SOD activity only slightly in wild-type and empty vector plants, but more strongly in 1B-OEX plants. SOD activity in 1B-siRNA plants was not induced by drought stress suggesting that TaZFP1B is needed for *SOD* up-regulation. Similar results were observed for the two other antioxidant enzymes (APX and CAT) (Fig. 5c to f).

Non-enzymatic antioxidants such as reduced glutathione (GSH) have been reported to play a significant role in the management of oxidative stress [68]. We assayed the total glutathione content by measuring reduced (GSH) and oxidized (GSSG) glutathione in leaf tissues. As shown in Fig. 5g, a higher amount of GSH was observed in 1B-OEX plants compared to the wild-type, empty vector and 1B-siRNA plants under well-watered conditions. After 7 days of drought stress, similar amounts of GSH were observed in wild-type, empty vector and 1B-siRNA plants, while 1B-OEX plants showed a higher amount of GSH. Under control conditions, the four plant types had similar GSSG levels. Drought stress caused a significant accumulation of GSSG in the four plants and this accumulation was highest in 1B-OEX plants (Fig. 5h). These results suggest that the antioxidant capacity is increased by TaZFP1B.

### Transcriptome modifications under well-watered conditions

**Fig. 6:**
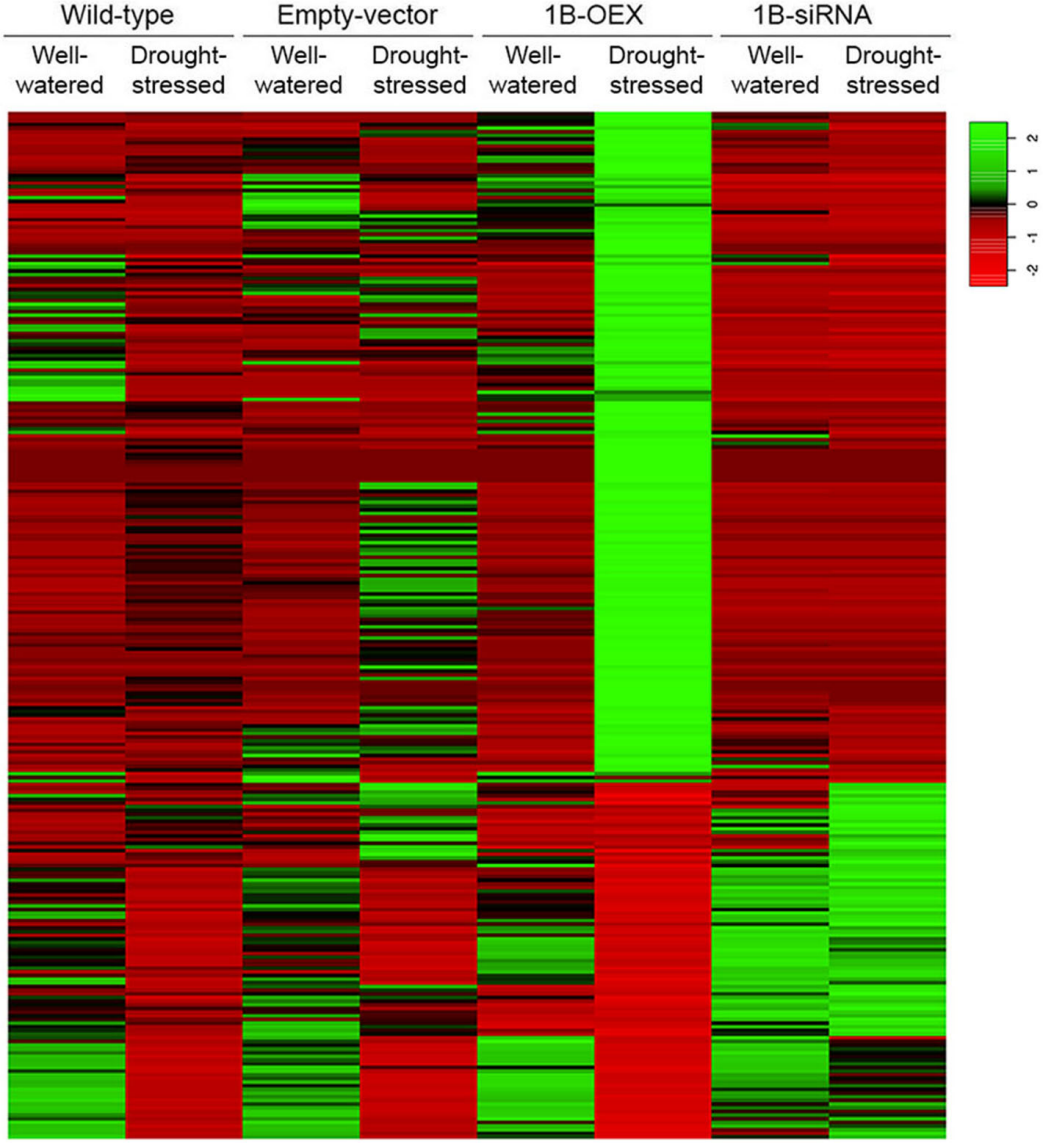
Heat map showing the differential gene expression. Plants were grown and treated as described in Fig. 2 except that drought stress was applied for 7 days, then RNA-Seq libraries were prepared and sequenced. The hierarchical clustering was generated using Spearman correlation coefficients of log2-transformed TPM expression values. The color scale indicates the expression levels (red, low expression; green, high expression). The 187 genes up-regulated at least five-fold in 1B-OEX plants under drought stress are listed in Table 4. The 96 genes down-regulated at least two-fold by TaZFP1B overexpression under drought stress are listed in Table 5

To better understand the function of TaZFP1B at the molecular level, eight mRNA-Seq libraries were sequenced to analyze the transcript profiles. To determine how the *TaZFP1B* transcript level affects gene expression, the mRNA profiles of 1B-OEX and 1B-siRNA plants were compared to that of wild-type plants, under well-watered or drought conditions. The global expression pattern was visualized by generating a heat map of the differentially regulated transcripts between the different types of plants (Fig. 6). We found that 27 transcripts were up-regulated by at least 5-fold in 1B-OEX well-watered plants (Table 2). These up-regulated genes encode proteins involved in transcription, calcium binding, stress response, oxidation-reduction, cell wall and membrane structure, transport, cell cycle and carbohydrate metabolism. Some of these proteins have been associated with abiotic stress responses.

**Table 2:** Genes up-regulated at least five-fold by *TaZFP1B* overexpression under well-watered conditions

Gene number #	Gene	Annotation	Fold change
**Transcription factor**			
1	TRIAE_CS42_3DL_TGACv1_249236_AA0842580.1	Zinc finger homeodomain protein 5	55,9
2	TRIAE_CS42_2AS_TGACv1_112557_AA0340950.6	Eukaryotic translation initiation factor	53,9
3	TRIAE_CS42_3AL_TGACv1_196955_AA0663930.1	Transcription factor PCF7	5,3
**Calcium binding proteins**			
4	TRIAE_CS42_5BL_TGACv1_405470_AA1328020.1	Calreticulin	5,3
**Stress-related proteins**			
5	TRIAE_CS42_5AL_TGACv1_376571_AA1238590.1	DEAD-box ATP-dependent RNA helicase	> 100
6	TRIAE_CS42_4AL_TGACv1_293117_AA1000210.1	Aspartic protease	11,7
7	TRIAE_CS42_6AL_TGACv1_474009_AA1533790.1	Lysine-specific demethylase JMJ706-like	9,4
8	TRIAE_CS42_7AS_TGACv1_569033_AA1805230.1	ACT domain repeat protein	7,3
9	TRIAE_CS42_3B_TGACv1_222967_AA0774260.2	Chitinase 10	6,6
10	TRIAE_CS42_3DS_TGACv1_272269_AA0918140.2	Disease resistance protein RPM1	5,2
11	TRIAE_CS42_4AS_TGACv1_307024_AA1016270.3	BTB/POZ domain-containing protein NPY4	5,1
12	TRIAE_CS42_5AL_TGACv1_376986_AA1242950.1	Glutathione S-transferase	5,0
**Oxidation-reduction process**			
13	TRIAE_CS42_3AL_TGACv1_194817_AA0640010.2	Aldo-keto reductase	39,0
14	TRIAE_CS42_5AL_TGACv1_376972_AA1242830.1	Alcohol dehydrogenase ADH2H	10,8
15	TRIAE_CS42_2BL_TGACv1_129634_AA0391150.1	DMR6-like oxygenase	7,1
**Cell wall and membrane structure**			
16	TRIAE_CS42_7BS_TGACv1_592358_AA1936370.2	Profilin actin binding protein	38,5
17	TRIAE_CS42_U_TGACv1_694116_AA2161830.1	Xyloglucan endotransglucosylase/hydrolase	6,5
**Transporters**			
18	TRIAE_CS42_6BL_TGACv1_500270_AA1602490.3	TIC 20 protein	6,8
19	TRIAE_CS42_7DS_TGACv1_621736_AA2024850.1	WAT1-related protein	6,2
20	TRIAE_CS42_3AL_TGACv1_195744_AA0653320.1	Bidirectional sugar transporter SWEET	5,9
**Cell cycle**			
21	TRIAE_CS42_4DS_TGACv1_362060_AA1176230.3	Structural maintenance of chromosomes (SMC) protein	> 100
22	TRIAE_CS42_2BL_TGACv1_130589_AA0414320.3	Protein laz1	> 100
**Carbohydrate metabolism-related proteins**			
23	TRIAE_CS42_7BL_TGACv1_578713_AA1899080.1	Glucan endo-1,3-beta-glucosidase	> 100
**Others**			
24	TRIAE_CS42_U_TGACv1_641461_AA2095670.2	Glutamate receptor interacting protein	> 100
25	TRIAE_CS42_5BL_TGACv1_406408_AA1345220.1	Ankyrin repeat containing protein	> 100
26	TRIAE_CS42_6DL_TGACv1_526505_AA1685690.2	F-box domain, cyclin-like domain containing protein	31,0
27	TRIAE_CS42_5DL_TGACv1_434415_AA1435460.1	TolB-like domain containing protein	7,5

Fold change, 1B-OEX to wild-type ratio. The gene numbers (#) are used in the text

Although a 5-fold induction in gene expression provides confidence that the genes are regulated by TaZFP1B, genes up-regulated between one and five-fold in 1B-OEX plants may nevertheless play a significant role in stress tolerance. For example, genes encoding enzymes known for their role in ROS scavenging (Additional file 3: Table S1) are of particular interest: SOD increases 2.5-fold, APX increases 2.1-fold and CAT increases 1.8-fold. Furthermore, overexpression of *TaZFP1B* also down-regulates 11 transcripts by at least 2-fold (Table 3).

**Table 3:** Genes down-regulated at least two-fold by *TaZFP1B* overexpression under well-watered conditions

Gene number #	Gene	Annotation	Fold change
**Transcription factor**			
28	TRIAE_CS42_1AL_TGACv1_001758_AA0034810.2	G-box binding factor	2,8
**Transporter**			
29	TRIAE_CS42_3B_TGACv1_223624_AA0784820.1	Protein DETOXIFICATION	10,7
30	TRIAE_CS42_U_TGACv1_642488_AA2118360.7	Copper transporter CT1	10,4
31	TRIAE_CS42_5BL_TGACv1_404850_AA1312820.1	ZINC INDUCED FACILITATOR	2,8
**Carbohydrate metabolism-related proteins**			
32	TRIAE_CS42_1DS_TGACv1_081598_AA0261790.1	Malonyl-coenzyme A:anthocyanin 3-O-glucoside-6″-O-malonyltransferase-like	47,9
**Others**			
33	TRIAE_CS42_3B_TGACv1_226496_AA0817320.2	Unknown protein	4,5
34	TRIAE_CS42_7BS_TGACv1_593046_AA1947680.1	Unknown protein	4,3
35	TRIAE_CS42_3B_TGACv1_221867_AA0752050.2	Bark storage protein A-like	2,9
36	TRIAE_CS42_7DS_TGACv1_622168_AA2034370.3	Protein REVEILLE	2,4
37	TRIAE_CS42_6BL_TGACv1_501185_AA1614740.1	Unknown protein	2,3
38	TRIAE_CS42_3B_TGACv1_221388_AA0739090.1	Unknown protein	2,0

Fold change, wild-type to 1B-OEX ratio. The gene numbers (#) are used in the text

### Transcriptome modifications under drought stress

To identify molecular pathways by which *TaZFP1B* confers drought tolerance in wheat, transcript levels were compared between 1B-OEX and wild-type plants under drought stress. Our analyses revealed that overexpression of *TaZFP1B* modifies the expression of many new genes during drought stress compared to the other three types of plants (Fig. 6). We found 187 transcripts that were up-regulated by at least 5-fold (Table 4) in drought-treated 1B-OEX plants compared to wild-type plants. Among these are genes encoding proteins involved in transcription, signal transduction, stress responses, oxidation-reduction processes, cell wall and membrane structure, cell cycle, transport, protein post-translational modifications, carbohydrate, fatty acid and nitrogen metabolisms, and other metabolisms. Of interest are the data on genes associated with ROS. Genes encoding enzymes that stimulate ROS production, for example NADPH oxidase (#143) and galactose oxidase (#149), are up-regulated concomitantly with genes encoding ROS-scavenging enzymes such as peroxidases (#137, #141, #142) and thioredoxin (#153). A host of genes known for their role in stress response and tolerance are also up-regulated (Table 4): genes encoding dehydrins (#91, #95, #100, #114), cold-responsive proteins COR14a (#69), glucan endo-1,3-beta-glucosidase (#182), repeat domain proteins (#92, #102, #125–127), NADP-dependent malic enzyme (#183), E3 ubiquitin-protein ligase (#178, #179), glycine or hydroxyproline-rich proteins (#113, #159) and genes involved in programmed cell death (#148, #160, #164, #165). Moreover, TaZFP1B up-regulates several genes encoding enzymes involved in cell wall modifications such as xyloglucan endotransglucosylase/hydrolase (#154) and pectinesterase (#155), and genes encoding other proteins associated with cell wall remodeling such as expansin (#156), remorin (#157) and WAX2 (#158). Several genes involved in carbohydrate and fatty acid metabolisms are also up-regulated (#182–195). On the other hand, 96 transcripts are down-regulated by at least 2-fold in drought-treated 1B-OEX plants compared to wild-type plants (Table 5). Many of these are genes involved in photosynthesis metabolism (#244–264). Together, these observations emphasize the role of TaZFP1B in transcriptional regulation under drought stress and suggest that TaZFP1B is a key regulator of stress-related genes which are important for drought stress and oxidative stress tolerance.

**Table 4:** Genes up-regulated at least five-fold by *TaZFP1B* overexpression under drought stress

Gene number #	Gene	Annotation	Fold change
**Transcription-related proteins**			
39	TRIAE_CS42_3B_TGACv1_223209_AA0778000.3	LSD1 zinc finger	> 100
40	TRIAE_CS42_5DL_TGACv1_434621_AA1438900.6	Scarecrow-like protein	> 100
41	TRIAE_CS42_7BS_TGACv1_592226_AA1933690.4	Transcriptional corepressor LEUNIG	> 100
42	TRIAE_CS42_3AS_TGACv1_212728_AA0702890.1	Transcriptional regulator RABBIT EARS	42,2
43	TRIAE_CS42_3AL_TGACv1_195838_AA0654650.3	Double-stranded RNA-binding protein 1	28,9
44	TRIAE_CS42_4DL_TGACv1_343075_AA1129010.3	Trihelix transcription factor GT-2	24,1
45	TRIAE_CS42_3AL_TGACv1_196955_AA0663930.1	Transcription factor PCF7	22,5
46	TRIAE_CS42_1DL_TGACv1_061611_AA0199920.1	DnaJ homolog subfamily B	20,5
47	TRIAE_CS42_6BS_TGACv1_514227_AA1657240.1	B-box zinc finger protein	19,7
48	TRIAE_CS42_2DL_TGACv1_159186_AA0534130.1	WRKY transcription factor 12	18,4
49	TRIAE_CS42_5BL_TGACv1_406061_AA1340040.1	CBFIVd-B4	18,4
50	TRIAE_CS42_2DL_TGACv1_159186_AA0534130.1	WRKY transcription factor	18,4
51	TRIAE_CS42_2AL_TGACv1_095147_AA0307480.1	Lateral organ boundaries transcription factor	16,4
52	TRIAE_CS42_5DL_TGACv1_435031_AA1445050.1	Ocs element-binding factor	14.2
53	TRIAE_CS42_2BS_TGACv1_147417_AA0482900.1	Zinc finger protein CONSTANS 15	15,8
54	TRIAE_CS42_1DL_TGACv1_062044_AA0207980.1	Transcription factor bHLH 112	15,1
55	TRIAE_CS42_7AL_TGACv1_558337_AA1792520.4	Zinc finger CCCH domain-containing protein	14,2
56	TRIAE_CS42_4DS_TGACv1_361293_AA1165150.6	BTB/POZ domain-containing protein	12,6
57	TRIAE_CS42_1DL_TGACv1_064769_AA0235650.2	DnaJ homolog subfamily C member	11,9
58	TRIAE_CS42_4AS_TGACv1_306719_AA1012420.1	Homeobox-leucine zipper protein HOX12	11,9
59	TRIAE_CS42_2AS_TGACv1_113089_AA0351070.5	Heterogeneous nuclear ribonucleoprotein 1	10,7
60	TRIAE_CS42_3DS_TGACv1_271642_AA0904520.1	Ethylene-responsive transcription factor CRF1	10,5
61	TRIAE_CS42_7DS_TGACv1_624521_AA2061810.2	Transcription factor bHLH 78	9,7
62	TRIAE_CS42_1DS_TGACv1_080414_AA0247520.3	DnaJ homolog subfamily B member	9,4
63	TRIAE_CS42_5DS_TGACv1_457055_AA1481570.1	Zinc finger protein C2H2 type	9,3
64	TRIAE_CS42_5BS_TGACv1_424687_AA1391330.5	Transcription factor bZIP	8,2
65	TRIAE_CS42_5BL_TGACv1_406061_AA1340050.1	Dehydration-responsive element-binding protein	7,4
66	TRIAE_CS42_1DS_TGACv1_080938_AA0255890.1	C3HC4 type zinc-finger (RING finger)	6,9
67	TRIAE_CS42_3B_TGACv1_224651_AA0799380.1	Lateral organ boundaries transcription factor	6,9
68	TRIAE_CS42_5DL_TGACv1_437778_AA1467310.5	Two-component response regulator	6,7
69	TRIAE_CS42_2DL_TGACv1_162669_AA0563320.1	Cold-responsive protein COR14a	6,2
70	TRIAE_CS42_5BS_TGACv1_424687_AA1391330.3	Transcription factor bZIP	5,9
71	TRIAE_CS42_4DL_TGACv1_345275_AA1152550.1	Homeobox-leucine zipper protein HOX13	5,7
72	TRIAE_CS42_7BS_TGACv1_592977_AA1946950.1	Transcription factor bHLH HEC2	5,6
73	TRIAE_CS42_7AS_TGACv1_569468_AA1816690.1	C2-C2 zinc finger	5,6
74	TRIAE_CS42_7BL_TGACv1_577432_AA1875300.1	VQ domain containing protein	5,5
75	TRIAE_CS42_5AL_TGACv1_375766_AA1226930.1	CBF IVd-A22	5,2
76	TRIAE_CS42_6DS_TGACv1_544607_AA1748880.1	B-box zinc finger protein	5,2
77	TRIAE_CS42_3AL_TGACv1_195166_AA0645630.3	Transcription factor bHLH87	5,1
**Calcium binding protein, kinase or phosphatase**			
78	TRIAE_CS42_7BS_TGACv1_594052_AA1955780.1	Phytosulfokine receptor 1	84,1
79	TRIAE_CS42_5DL_TGACv1_434773_AA1441380.1	LRR receptor-like serine/threonine-protein kinase	35,9
80	TRIAE_CS42_4DS_TGACv1_362249_AA1178220.1	Calmodulin CML2	14,8
81	TRIAE_CS42_7BL_TGACv1_577549_AA1878500.1	Serine/threonine protein kinase	14,0
82	TRIAE_CS42_2DS_TGACv1_177289_AA0572410.1	EF-Hand type domain containing protein	9,2
83	TRIAE_CS42_6DL_TGACv1_527174_AA1700020.3	Mitogen-activated protein kinase kinase kinase	7,6
84	TRIAE_CS42_7AL_TGACv1_559296_AA1799010.1	Receptor-like protein kinase	6,9
**Stress-related proteins**			
85	TRIAE_CS42_4AS_TGACv1_307024_AA1016270.3	BTB/POZ domain-containing protein NPY4	> 100
86	TRIAE_CS42_2AL_TGACv1_093753_AA0286070.3	Protein DJ-1 homolog D	> 100
87	TRIAE_CS42_U_TGACv1_641289_AA2090920.5	Topless-related protein 1	> 100
88	TRIAE_CS42_3DS_TGACv1_272269_AA0918140.2	Disease resistance protein RPM1	> 100
89	TRIAE_CS42_4AS_TGACv1_308773_AA1029400.2	Pathogenesis-related protein	> 100
90	TRIAE_CS42_7AS_TGACv1_569748_AA1823280.2	Cysteine synthase	86,2
91	TRIAE_CS42_5DL_TGACv1_433513_AA1415280.1	Dehydrin DHN2	73,3
92	TRIAE_CS42_4BS_TGACv1_328898_AA1095240.1	Pentatricopeptide repeat containing protein	67,2
93	TRIAE_CS42_2DL_TGACv1_163849_AA0564640.1	Wound induced protein	57,4
94	TRIAE_CS42_2AS_TGACv1_115694_AA0372890.2	Cytochrome P450 family protein	50,4
95	TRIAE_CS42_5BL_TGACv1_406032_AA1339460.1	Dehydrin DHN2	50,4
96	TRIAE_CS42_2AL_TGACv1_095873_AA0315400.1	Mediator of ABA-regulated dormancy 1	48,4
97	TRIAE_CS42_2DL_TGACv1_160571_AA0551860.1	Mediator of ABA-regulated dormancy 1	31,6
98	TRIAE_CS42_7BS_TGACv1_592018_AA1928290.1	Glutathione S-transferase	27,3
99	TRIAE_CS42_2DL_TGACv1_160490_AA0551070.1	Wound-responsive family protein	26,3
100	TRIAE_CS42_5AL_TGACv1_376309_AA1235150.1	Dehydrin DHN2	22,9
101	TRIAE_CS42_5AL_TGACv1_374112_AA1190770.1	Aspartic protease	22,7
102	TRIAE_CS42_4AL_TGACv1_290111_AA0981810.2	Tetratricopeptide repeat protein	17,4
103	TRIAE_CS42_7DL_TGACv1_602552_AA1960450.1	Tubby-like protein	17,0
104	TRIAE_CS42_6AS_TGACv1_486459_AA1561390.1	Auxin-responsive protein SAUR	16,9
105	TRIAE_CS42_2DL_TGACv1_158690_AA0524470.1	Late embryogenesis abundant protein	14,7
106	TRIAE_CS42_2DL_TGACv1_158690_AA0524470.1	Late embryogenesis abundant protein	14,6
107	TRIAE_CS42_4DL_TGACv1_343378_AA1133790.1	Defensin	14,1
108	TRIAE_CS42_5BL_TGACv1_404954_AA1316190.1	Cinnamoyl-CoA reductase	13,1
109	TRIAE_CS42_7AS_TGACv1_569113_AA1807500.2	BTB/POZ and MATH domain-containing protein	13,1
110	TRIAE_CS42_5DL_TGACv1_433130_AA1403020.1	Stress responsive A/B barrel domain-containing protein	12,9
111	TRIAE_CS42_U_TGACv1_640759_AA2072760.14	Cysteine proteinase superfamily protein	12,5
112	TRIAE_CS42_7AS_TGACv1_569033_AA1805230.1	ACT domain repeat protein	11,6
113	TRIAE_CS42_4BL_TGACv1_320342_AA1036020.1	Glycine-rich protein	11,3
114	TRIAE_CS42_5AL_TGACv1_376309_AA1235140.1	Dehydrin DHN1	11,2
115	TRIAE_CS42_4AL_TGACv1_293117_AA1000210.1	Aspartic protease	10,9
116	TRIAE_CS42_6BS_TGACv1_513372_AA1639240.1	F-box/kelch-repeat protein SKIP4	10,5
117	TRIAE_CS42_1BL_TGACv1_030562_AA0094080.1	Cytochrome P450 85A1	10,5
118	TRIAE_CS42_6DS_TGACv1_542552_AA1724150.1	Auxin-responsive protein SAUR71	10,4
119	TRIAE_CS42_5BL_TGACv1_406277_AA1343590.3	Cysteine proteinase superfamily protein	9,1
120	TRIAE_CS42_7DS_TGACv1_621863_AA2028000.1	Cytochrome c oxidase subunit 5B mitochondrial	8,9
121	TRIAE_CS42_5DL_TGACv1_434334_AA1434100.2	F-box/kelch-repeat protein	8,8
122	TRIAE_CS42_5DL_TGACv1_435810_AA1455180.2	Rhodanese-like domain-containing protein	8,1
123	TRIAE_CS42_5AL_TGACv1_376986_AA1242950.1	Glutathione S-transferase	7,8
124	TRIAE_CS42_7BS_TGACv1_591856_AA1923720.1	Aspartic protease	7,5
125	TRIAE_CS42_5DL_TGACv1_434499_AA1437000.1	Pentatricopeptide repeat-containing protein	7,2
126	TRIAE_CS42_6AL_TGACv1_471352_AA1507530.3	WD repeat-containing protein	6,1
127	TRIAE_CS42_6AL_TGACv1_471352_AA1507530.6	WD repeat-containing protein	6,1
128	TRIAE_CS42_3B_TGACv1_223361_AA0780920.2	Glutathione transferase GST	5,8
129	TRIAE_CS42_3AL_TGACv1_197123_AA0664910.3	Universal stress protein PHOS32	5,7
130	TRIAE_CS42_1BL_TGACv1_030249_AA0083800.1	Universal stress protein PHOS32	5,7
131	TRIAE_CS42_3AS_TGACv1_210945_AA0681930.1	AAA-protein family	5,5
132	TRIAE_CS42_1AL_TGACv1_001038_AA0023900.1	Universal stress protein PHOS	5,5
133	TRIAE_CS42_2AL_TGACv1_093314_AA0277190.2	Heat shock protein	5,3
134	TRIAE_CS42_3DL_TGACv1_249203_AA0841110.1	Abscisic stress-ripening protein	5,3
135	TRIAE_CS42_2AL_TGACv1_094123_AA0292860.2	Elicitor responsive gene	5,2
136	TRIAE_CS42_6AS_TGACv1_487227_AA1569190.1	Auxin-induced protein	5,0
**Oxidation-reduction process**			
137	TRIAE_CS42_2DS_TGACv1_178485_AA0596270.1	Peroxidase	44,7
138	TRIAE_CS42_4AL_TGACv1_290177_AA0982590.1	Alcohol dehydrogenase ADH1A	23,1
139	TRIAE_CS42_7BL_TGACv1_576933_AA1860390.1	Defensin	19,0
140	TRIAE_CS42_2AS_TGACv1_112531_AA0340030.1	Flavin-containing monooxygenase	18,9
141	TRIAE_CS42_2DS_TGACv1_177840_AA0585640.1	Peroxidase	18,6
142	TRIAE_CS42_2BS_TGACv1_146806_AA0473250.1	Peroxidase	15,9
143	TRIAE_CS42_5AL_TGACv1_377290_AA1245640.4	NADPH oxidase	9,9
144	TRIAE_CS42_2DL_TGACv1_160970_AA0555660.1	Protochlorophyllide reductase A	7,7
145	TRIAE_CS42_2AL_TGACv1_096139_AA0317380.1	Hyoscyamine 6-dioxygenase	7,4
146	TRIAE_CS42_5AL_TGACv1_376972_AA1242830.1	Alcohol dehydrogenase ADH2H	6,9
147	TRIAE_CS42_2DL_TGACv1_158391_AA0517380.1	FAD-dependent urate hydroxylase-like	6,4
148	TRIAE_CS42_2AL_TGACv1_096158_AA0317510.1	Polyamine oxidase 4	6,0
149	TRIAE_CS42_2AL_TGACv1_096643_AA0320430.1	Galactose oxidase	5,9
150	TRIAE_CS42_7AS_TGACv1_570735_AA1840120.1	Thiosulfate sulfurtransferase	5,7
151	TRIAE_CS42_1AL_TGACv1_002876_AA0045790.1	Gibberellin 20 oxidase 2	5,5
152	TRIAE_CS42_3AL_TGACv1_194843_AA0640510.2	Selenium-binding protein	5,4
153	TRIAE_CS42_1BL_TGACv1_031952_AA0123300.2	Thioredoxin protein	5,0
**Cell wall and membrane structure**			
154	TRIAE_CS42_U_TGACv1_694116_AA2161830.1	Xyloglucan endotransglucosylase/hydrolase	> 100
155	TRIAE_CS42_1AL_TGACv1_000357_AA0009840.1	Pectinesterase	26,8
156	TRIAE_CS42_5AS_TGACv1_393897_AA1277020.1	Expansin-B3	19,0
157	TRIAE_CS42_5DL_TGACv1_433202_AA1405370.1	Remorin	8,2
158	TRIAE_CS42_6DL_TGACv1_527336_AA1702400.1	Protein WAX2	7,3
159	TRIAE_CS42_5AS_TGACv1_394403_AA1280080.3	Hydroxyproline-rich glycoprotein family protein	7,2
**Cell cycle**			
160	TRIAE_CS42_2BL_TGACv1_130589_AA0414320.3	Protein laz1	> 100
161	TRIAE_CS42_U_TGACv1_640759_AA2072760.14	Cysteine proteinases superfamily protein	12,5
162	TRIAE_CS42_5BL_TGACv1_406277_AA1343590.3	Cysteine proteinases superfamily protein	9,1
163	TRIAE_CS42_3DL_TGACv1_249314_AA0845060.3	Mitotic spindle checkpoint protein MAD1	7,4
164	TRIAE_CS42_1BL_TGACv1_030567_AA0094200.2	Protein LOL1	7,3
165	TRIAE_CS42_5AL_TGACv1_375766_AA1226930.1	Protein LOL1	6,6
166	TRIAE_CS42_2DS_TGACv1_177975_AA0588450.1	Cyclin-D2–2	5,1
167	TRIAE_CS42_3DS_TGACv1_271852_AA0909480.1	Cell division cycle-associated protein	5,0
**Transporters**			
168	TRIAE_CS42_3DL_TGACv1_250015_AA0860390.2	GABA transporter 1	> 100
169	TRIAE_CS42_3DL_TGACv1_249597_AA0852170.3	Bidirectional sugar transporter SWEET	86,3
170	TRIAE_CS42_2AL_TGACv1_093880_AA0288670.1	Non-specific lipid-transfer protein	16,2
171	TRIAE_CS42_5DL_TGACv1_432929_AA1394800.5	Heavy metal-associated isoprenylated protein 32	10,4
172	TRIAE_CS42_1DL_TGACv1_061859_AA0204490.1	Non-specific lipid-transfer protein	8,1
173	TRIAE_CS42_3AL_TGACv1_195744_AA0653320.1	Bidirectional sugar transporter SWEET	6,5
174	TRIAE_CS42_6DL_TGACv1_526901_AA1694680.2	Bidirectional sugar transporter SWEET	6,1
175	TRIAE_CS42_1AL_TGACv1_003689_AA0051080.1	Non-specific lipid-transfer protein	6,0
176	TRIAE_CS42_1DL_TGACv1_061102_AA0185280.1	Non-specific lipid-transfer protein	5,8
177	TRIAE_CS42_7BS_TGACv1_593410_AA1951130.1	Non-specific lipid-transfer protein	5,6
**Post-translational protein modification**			
178	TRIAE_CS42_4DS_TGACv1_361658_AA1171150.4	E3 ubiquitin-protein ligase ARI1	9,1
179	TRIAE_CS42_3DS_TGACv1_272908_AA0926480.3	E3 ubiquitin-protein ligase	8,4
180	TRIAE_CS42_1AL_TGACv1_000329_AA0009100.2	Ubiquitin conjugation factor E4 protein	7,9
181	TRIAE_CS42_7BL_TGACv1_578537_AA1896750.2	Dolichyl-diphosphooligosaccharide protein glycosyltransferase subunit DAD1	7,1
**Carbohydrate metabolism-related proteins**			
182	TRIAE_CS42_7BL_TGACv1_578713_AA1899080.1	Glucan endo-1,3-beta-glucosidase 3	> 100
183	TRIAE_CS42_3AL_TGACv1_194492_AA0634060.2	NADP-dependent malic enzyme	> 100
184	TRIAE_CS42_3DL_TGACv1_249576_AA0851790.4	Beta-galactosidase	79,1
185	TRIAE_CS42_5DL_TGACv1_436125_AA1458320.1	Beta-glucosidase	53,9
186	TRIAE_CS42_2AS_TGACv1_112777_AA0345080.1	Transketolase	32,4
187	TRIAE_CS42_5BL_TGACv1_405377_AA1326060.1	Phosphoglycerate mutase-like protein	12,8
188	TRIAE_CS42_2BL_TGACv1_131851_AA0432880.2	Aldose 1-epimerase	10,2
189	TRIAE_CS42_3B_TGACv1_222532_AA0766530.4	ATP synthase subunit beta	6,6
190	TRIAE_CS42_2DL_TGACv1_158102_AA0509180.1	Phenolic glucoside malonyltransferase	6,2
191	TRIAE_CS42_5AS_TGACv1_392538_AA1260340.7	GDP-L-galactose phosphorylase	6,1
192	TRIAE_CS42_1DL_TGACv1_061598_AA0199620.1	Beta-galactosidase 7	4,7
**Fatty acid metabolism-related proteins**			
193	TRIAE_CS42_3AS_TGACv1_210886_AA0680800.3	Sphingosine-1-phosphate lyase	> 100
194	TRIAE_CS42_1AS_TGACv1_019138_AA0061500.2	GDSL esterase/lipase	8,8
195	TRIAE_CS42_1BL_TGACv1_030780_AA0100660.2	Phospholipase D	5,4
**Nitrogen metabolism-related proteins**			
196	TRIAE_CS42_4AS_TGACv1_307728_AA1023060.1	Glutamine synthetase	6,4
**Others**			
197	TRIAE_CS42_4DS_TGACv1_362426_AA1179710.6	Cyclin-like F-box domain containing protein	> 100
198	TRIAE_CS42_1BS_TGACv1_050119_AA0167640.2	Unknown protein	> 100
199	TRIAE_CS42_3AS_TGACv1_211314_AA0688450.2	WPP domain-interacting tail-anchored protein	> 100
200	TRIAE_CS42_5DL_TGACv1_434415_AA1435460.1	TolB-like domain containing protein	94,1
201	TRIAE_CS42_6DL_TGACv1_528348_AA1713440.1	Unknown protein	84,4
202	TRIAE_CS42_5BL_TGACv1_406843_AA1350500.1	Unknown protein	58,7
203	TRIAE_CS42_5DL_TGACv1_433372_AA1411340.1	Tryptophan synthase alpha chain-like	52,7
204	TRIAE_CS42_1AS_TGACv1_019383_AA0065910.3	Isovaleryl-CoA dehydrogenase	39,6
205	TRIAE_CS42_4DL_TGACv1_343143_AA1130470.1	Unknown protein	27,7
206	TRIAE_CS42_5DL_TGACv1_435042_AA1445250.2	Obg-like ATPase 1	27,1
207	TRIAE_CS42_1AS_TGACv1_019803_AA0071620.1	Cyclin-like F-box domain containing protein	23,5
208	TRIAE_CS42_2BL_TGACv1_130998_AA0421050.2	Peptidylprolyl isomerase	17,2
209	TRIAE_CS42_3DL_TGACv1_250372_AA0867150.1	Unknown protein	15,9
210	TRIAE_CS42_3AL_TGACv1_195382_AA0648850.1	Unknown protein	13,9
211	TRIAE_CS42_4BL_TGACv1_322190_AA1069510.1	Anthocyanidin 3-O-glucosyltransferase	12,2
212	TRIAE_CS42_4BL_TGACv1_321444_AA1060550.2	Elongator complex protein 6	10,8
213	TRIAE_CS42_5DL_TGACv1_433709_AA1420080.1	Nitrile-specifier protein 1	10,1
214	TRIAE_CS42_5AL_TGACv1_375015_AA1213930.1	Actin-depolymerizing factor 10	9,7
215	TRIAE_CS42_7DL_TGACv1_602612_AA1962750.1	Arogenate dehydrogenase 2	9,4
216	TRIAE_CS42_3DL_TGACv1_249368_AA0846860.1	Peptidase C1A, papain family protein	7,5
217	TRIAE_CS42_5AL_TGACv1_375655_AA1225200.2	deSI-like protein	7,3
218	TRIAE_CS42_3B_TGACv1_224606_AA0798680.1	Aromatic-ring hydroxylase domain containing protein	7,1
219	TRIAE_CS42_5BL_TGACv1_404868_AA1313220.1	Nitrile-specifier protein 1	5,9
220	TRIAE_CS42_4DL_TGACv1_343042_AA1128220.1	Golgin subfamily A member	5,8
221	TRIAE_CS42_1DL_TGACv1_061281_AA0191130.2	Plant UBX domain-containing protein 10	5,6
222	TRIAE_CS42_1AL_TGACv1_000217_AA0006430.1	Putative protein of unknown function (DUF640)	5,4
223	TRIAE_CS42_2AL_TGACv1_092997_AA0269140.3	Imidazoleglycerol-phosphate dehydratase	5,4
224	TRIAE_CS42_5BL_TGACv1_404429_AA1299570.1	Protein WVD2	5,2
225	TRIAE_CS42_5AL_TGACv1_376738_AA1240450.1	F-box domain, cyclin-like domain containing protein	5,0

Fold change, 1B-OEX to wild-type ratio. The gene numbers (#) are used in the text

**Table 5:** Genes down-regulated at least two-fold by *TaZFP1B* overexpression under drought stress

Gene number #	Gene	Annotation	Fold change
**Transcription factor**			
226	TRIAE_CS42_1AL_TGACv1_001758_AA0034810.2	G-box binding factor	5,0
227	TRIAE_CS42_4DS_TGACv1_361864_AA1173710.2	CCR4-NOT transcription complex subunit 3	3,0
228	TRIAE_CS42_4AS_TGACv1_307339_AA1019660.8	Scarecrow-like protein	2,4
**Calcium binding protein, kinase or phosphatase**			
229	TRIAE_CS42_5BS_TGACv1_423377_AA1375480.3	Dual specificity protein kinase shkD-like	8,5
230	TRIAE_CS42_4BL_TGACv1_320912_AA1051490.5	Phosphoinositide phosphatase SAC2	4,5
231	TRIAE_CS42_4DS_TGACv1_361621_AA1170770.1	Type IV inositol polyphosphate 5-phosphatase 11	3,0
232	TRIAE_CS42_7DL_TGACv1_603289_AA1980210.3	Haloacid dehalogenase-like hydrolase	2,9
233	TRIAE_CS42_6DL_TGACv1_526671_AA1689500.1	Calcium sensing receptor	2,6
**Stress-related proteins**			
234	TRIAE_CS42_U_TGACv1_645365_AA2144110.3	Protein argonaute	15,4
235	TRIAE_CS42_2AL_TGACv1_093339_AA0277990.3	Pentatricopeptide repeat (PPR-like) superfamily protein	6,2
236	TRIAE_CS42_5DL_TGACv1_434794_AA1441820.1	Disease resistance protein RPM1	3,5
237	TRIAE_CS42_7BL_TGACv1_579887_AA1910750.10	Spermidine synthase	2,9
238	TRIAE_CS42_5DS_TGACv1_456750_AA1477470.2	Chloroplast stem-loop binding protein of 41 kDa	2,9
239	TRIAE_CS42_5AS_TGACv1_393102_AA1268440.1	Chloroplast stem-loop binding protein of 41 kDa	2,8
240	TRIAE_CS42_2AS_TGACv1_112253_AA0334170.1	Tetratricopeptide repeat containing protein	2,6
241	TRIAE_CS42_2DL_TGACv1_158673_AA0524320.3	Zeaxanthin epoxidase	2,0
**Oxidation-reduction process**			
242	TRIAE_CS42_7DL_TGACv1_603859_AA1990270.1	NAD(P) H dehydrogenase (quinone) FQR1-like 1	14,6
243	TRIAE_CS42_6AL_TGACv1_472100_AA1517740.2	(+)-neomenthol dehydrogenase	3,3
**Photosynthesis related proteins**			
244	TRIAE_CS42_4BS_TGACv1_327886_AA1077790.6	Ribulose bisphosphate carboxylase/oxygenase activase A	18,2
245	TRIAE_CS42_1AS_TGACv1_019302_AA0064620.1	PGR5-like protein 1A	5,0
246	TRIAE_CS42_2AL_TGACv1_094760_AA0302740.1	Photosystem I subunit O	3,8
247	TRIAE_CS42_3DL_TGACv1_253211_AA0893780.1	Chlorophyllide a oxygenase, chloroplastic	2,9
248	TRIAE_CS42_2DS_TGACv1_177171_AA0567560.1	Ribulose bisphosphate carboxylase small chain	2,8
249	TRIAE_CS42_3DL_TGACv1_250162_AA0863350.3	Carbonic anhydrase	2,8
250	TRIAE_CS42_2BL_TGACv1_130248_AA0407140.1	Protein STAY-GREEN LIKE	2,7
251	TRIAE_CS42_U_TGACv1_642994_AA2125780.1	Photosystem II 5 kD protein	2,7
252	TRIAE_CS42_3B_TGACv1_222152_AA0758310.2	Carbonic anhydrase	2,6
253	TRIAE_CS42_4DL_TGACv1_342533_AA1116020.1	Photosystem II subunit X	2,6
254	TRIAE_CS42_4AL_TGACv1_290053_AA0980930.6	Ribulose bisphosphate carboxylase/oxygenase activase A	2,5
255	TRIAE_CS42_4DS_TGACv1_361664_AA1171210.2	Ribulose bisphosphate carboxylase/oxygenase activase A,	2,5
256	TRIAE_CS42_6AS_TGACv1_486261_AA1559010.1	Photosystem II 5 kD protein	2,3
257	TRIAE_CS42_2BL_TGACv1_129762_AA0395100.1	Photosystem I reaction center subunit	2,2
258	TRIAE_CS42_5BL_TGACv1_404244_AA1291940.1	Photosystem I reaction center subunit	2,2
259	TRIAE_CS42_2AL_TGACv1_093154_AA0273500.1	Ribulose bisphosphate carboxylase/oxygenase activase	2,2
260	TRIAE_CS42_5AL_TGACv1_375138_AA1216740.1	Chlorophyll a-b binding protein	2,1
261	TRIAE_CS42_2DL_TGACv1_162716_AA0563420.2	Probable plastid-lipid-associated protein 7	2,0
262	TRIAE_CS42_5BL_TGACv1_405399_AA1326490.1	Chlorophyll a-b binding protein	2,0
263	TRIAE_CS42_3AS_TGACv1_210772_AA0678660.5	SCAR-like protein 2	2,0
264	TRIAE_CS42_4BS_TGACv1_329104_AA1097840.1	Serine transhydroxymethyltransferase	2,0
**Transporter**			
265	TRIAE_CS42_U_TGACv1_642488_AA2118360.7	Copper transporter CT1	10,4
266	TRIAE_CS42_1DL_TGACv1_061382_AA0193640.2	Protein YIPF	5,7
267	TRIAE_CS42_3B_TGACv1_223624_AA0784820.1	Protein DETOXIFICATION	3,5
268	TRIAE_CS42_5DL_TGACv1_435599_AA1452500.1	Mitochondrial substrate carrier family protein C	2,6
269	TRIAE_CS42_5BS_TGACv1_423469_AA1377600.2	CSC1-like protein	2,4
270	TRIAE_CS42_5BL_TGACv1_404850_AA1312820.1	ZINC INDUCED FACILITATOR	2,2
271	TRIAE_CS42_7AL_TGACv1_557283_AA1779210.1	ABC transporter B family	2,2
**Cell cycle**			
272	TRIAE_CS42_2DL_TGACv1_160031_AA0545750.1	Cyclin-P1–1	15,8
**Carbohydrate metabolism-related proteins**			
273	TRIAE_CS42_6DS_TGACv1_542696_AA1728130.1	Glycosyltransferase	9,3
274	TRIAE_CS42_3DL_TGACv1_249217_AA0841740.2	Sedoheptulose-1,7-bisphosphatase	2,9
275	TRIAE_CS42_1DS_TGACv1_081598_AA0261790.1	Malonyl-coenzyme A:anthocyanin 3-O-glucoside-6″-O-malonyltransferase	2,5
276	TRIAE_CS42_2AL_TGACv1_093283_AA0276660.3	Glyceraldehyde-3-phosphate dehydrogenase	2,4
277	TRIAE_CS42_2DL_TGACv1_158386_AA0517110.1	Glyceraldehyde-3-phosphate dehydrogenase	2,0
**Nitrogen metabolism-related proteins**			
278	TRIAE_CS42_2DL_TGACv1_161369_AA0558280.2	Glutamine synthetase	5,2
279	TRIAE_CS42_U_TGACv1_640900_AA2078630.2	Glutamine synthetase	3,0
**Others**			
280	TRIAE_CS42_1BS_TGACv1_049885_AA0163610.1	Unknown protein	26,1
281	TRIAE_CS42_4BS_TGACv1_330248_AA1106770.1	Root phototropism protein 2	25,0
282	TRIAE_CS42_7DL_TGACv1_605944_AA2008620.1	Unknown protein	14,8
283	TRIAE_CS42_7BL_TGACv1_580679_AA1915040.1	Unknown protein	10,4
284	TRIAE_CS42_6BL_TGACv1_501185_AA1614740.1	Unknown protein	9,2
285	TRIAE_CS42_2AL_TGACv1_094031_AA0291380.1	S-norcoclaurine synthase	7,6
286	TRIAE_CS42_1AL_TGACv1_003073_AA0047460.1	Unknown protein	6,9
287	TRIAE_CS42_2BL_TGACv1_130918_AA0419860.1	Unknown protein	5,6
288	TRIAE_CS42_7DS_TGACv1_622168_AA2034370.3	Protein REVEILLE	5,2
289	TRIAE_CS42_3B_TGACv1_226496_AA0817320.2	Unknown protein	5,2
290	TRIAE_CS42_5DS_TGACv1_457673_AA1488760.1	Unknown protein	5,2
291	TRIAE_CS42_3B_TGACv1_221867_AA0752050.2	Bark storage protein A-like	4,9
292	TRIAE_CS42_6AS_TGACv1_485239_AA1541330.2	Unknown protein	4,5
293	TRIAE_CS42_7DS_TGACv1_625467_AA2065230.1	Unknown protein	4,1
294	TRIAE_CS42_3B_TGACv1_223220_AA0778090.1	Unknown protein	3,7
295	TRIAE_CS42_3AS_TGACv1_212545_AA0701630.2	Unknown function	3,5
296	TRIAE_CS42_3B_TGACv1_224639_AA0799250.6	Phosphatidate cytidylyltransferase	3,3
297	TRIAE_CS42_6AL_TGACv1_472100_AA1517740.2	(+)-neomenthol dehydrogenase-like	3,3
298	TRIAE_CS42_3B_TGACv1_224639_AA0799250.6	Phosphatidate cytidylyltransferase	3,3
299	TRIAE_CS42_5DL_TGACv1_434212_AA1431710.1	Unknown protein	3,3
300	TRIAE_CS42_3B_TGACv1_220931_AA0724270.1	Carboxyl-terminal-processing peptidase 1	3,2
301	TRIAE_CS42_3AL_TGACv1_194142_AA0627380.1	Unknown protein	3,2
302	TRIAE_CS42_6DL_TGACv1_527961_AA1710260.1	Unknown protein	2,9
303	TRIAE_CS42_3B_TGACv1_224332_AA0795340.1	Peptidase family M48 family protein	2,7
304	TRIAE_CS42_7BL_TGACv1_578255_AA1892020.2	(S)-coclaurine N-methyltransferase-like	2,7
305	TRIAE_CS42_7DL_TGACv1_604164_AA1994640.2	Unknown protein	2,7
306	TRIAE_CS42_3B_TGACv1_221388_AA0739090.1	Unknown protein	2,7
307	TRIAE_CS42_1AL_TGACv1_000467_AA0012810.1	Unknown protein	2,6
308	TRIAE_CS42_U_TGACv1_641221_AA2088730.1	Unknown protein	2,5
309	TRIAE_CS42_5BL_TGACv1_405011_AA1317790.1	Unknown protein	2,4
310	TRIAE_CS42_2BL_TGACv1_132610_AA0438610.1	Aminomethyltransferase	2,3
311	TRIAE_CS42_7BS_TGACv1_593046_AA1947680.1	Unknown protein	2,3
312	TRIAE_CS42_1DS_TGACv1_080446_AA0248140.1	Unknown protein	2,3
313	TRIAE_CS42_4DL_TGACv1_342977_AA1126610.1	Unknown protein	2,2
314	TRIAE_CS42_1AS_TGACv1_019551_AA0068040.1	Unknown protein	2,2
315	TRIAE_CS42_6BL_TGACv1_499775_AA1591420.1	Unknown protein	2,2
316	TRIAE_CS42_6BL_TGACv1_499688_AA1589190.1	Unknown protein	2,1
317	TRIAE_CS42_6BS_TGACv1_514327_AA1658410.1	Unknown protein	2,1
318	TRIAE_CS42_2DL_TGACv1_158386_AA0517100.1	Unknown protein	2,1
319	TRIAE_CS42_2BL_TGACv1_129404_AA0382260.1	Farnesyl pyrophosphate synthetase	2,1
320	TRIAE_CS42_4BS_TGACv1_329104_AA1097840.1	Serine transhydroxymethyltransferase 1	2,0
321	TRIAE_CS42_5DL_TGACv1_433432_AA1413030.1	Unknown protein	2,0

Fold change, wild-type to 1B-OEX ratio. The gene numbers (#) are used in the text

## Discussion

### The novel BSMV expression system allows functional gene characterization in wheat

Functional characterization in wheat is well-known as being more difficult to achieve than in model systems such as *Arabidopsis thaliana*. Furthermore, characterizing a crop gene in a heterologous system brings a host of questions that cannot be readily answered, and data interpretation cannot always be translated to the crop species. In this study, we demonstrate that the BSMV system allows for easy, fast and efficient gene characterization directly in wheat, an important crop species. Using the 4-component BSMV system that we have developed for VOX and the existing 3-component BSMV system for VIGS, we here show that the TaZFP1B transcription factor is required for oxidative and drought stress tolerance in wheat.

Drought is a major abiotic factor limiting growth and crop productivity worldwide. Improving drought tolerance in crops is an important consideration for agriculture sustainability, especially since climate change is expected to exacerbate the occurrence and severity of drought periods. From previous studies, we identified the C2H2-type zinc finger member *TaZFP1B* (previously named *TaZFP2*) as the gene most strongly up-regulated by various abiotic stresses (aluminum, high light, anoxia, H_2_O_2_ and drought) [60, 61]. Our current study demonstrates that overexpression of *TaZFP1B* does not cause growth reduction under normal growth conditions compared to other drought-associated transcription factors such as CBFs [69, 70]. Plants overexpressing *TaZFP1B* are more tolerant to drought while plants underexpressing *TaZFP1B* are more sensitive. The 1B-OEX plants have improved phenotypic parameters such as relative water content, dry matter production, shoot length, leaf width, survival rate and seed yield per spike compared to wild-type plants. This positive effect on growth might be mediated via a phytosulfokine receptor (#78) since overexpression *AtPSKR1* improves growth in Arabidopsis [71].

**Fig. 7:**
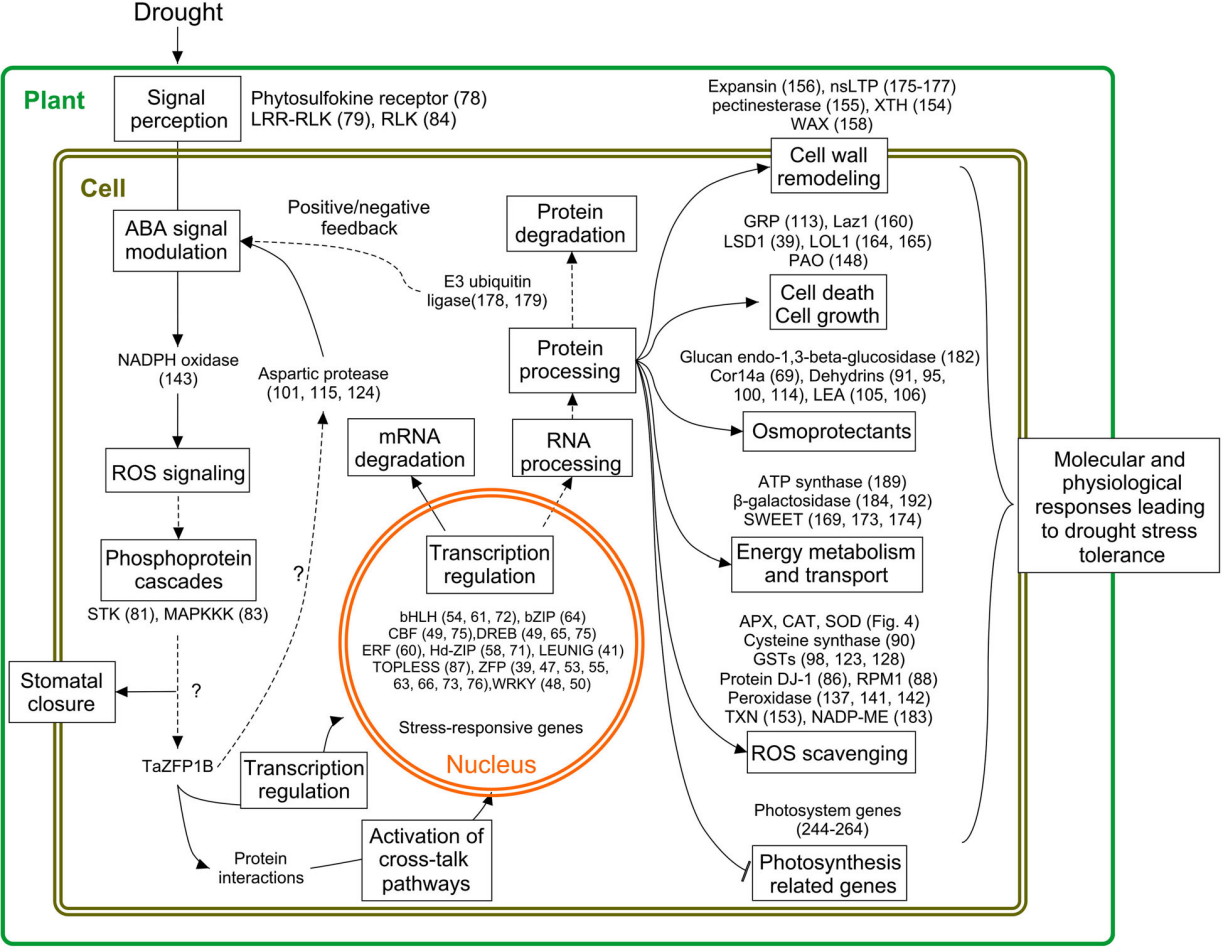
Summary of major changes in 1B-OEX plants and putative signaling pathways involved in drought tolerance. Solid lines indicate single-step reactions, and dashed lines indicate multi-step reactions. ABA, abscisic acid; APX, ascorbate peroxidase; bHLH, basic helix-loop-helix,; bZIP, basic leucine-zipper; CAT, catalase; CBF, core binding factor; COR, cold-regulated genes; DJ-1, protein deglycase DJ-1, DREB, dehydration-responsive element binding; ERF, ethylene response factor; GST, glutathione S-transferase; GRP, glycine-rich protein; Hd-ZIP, homeodomain-leucine zipper; HSP, heat shock proteins; Laz1, Lazarus 1; LEA, late embryogenesis abundant; LRR-RLK, leucine-rich repeats receptor-like kinase; LOL1, lsd one like 1; LSD1, lesion simulating disease 1; MAPKKK, mitogen-activated protein kinase kinase kinase; NADP-ME, NADP-dependent malic enzyme; nsLTP, non-specific lipid transfer protein; PAO, polyamine oxidase; SOD, superoxide dismutase; STK, serine/threonine kinase; ROS, reactive oxygen species; RUBISCO, ribulose bisphosphate carboxylase/oxygenase; TXN, thioredoxin protein; WAX; WRKY, transcription factor containing a highly conserved WRKY domain; and XTH, xyloglucan endotransglucosylase/hydrolase and ZFP, zinc finger protein;. Numbers refer to the corresponding genes in Tables 2, 3, 4 and 5

The transcriptome profiling experiments performed in this study revealed that TaZFP1B regulates a collection of transcripts involved in stress response and tolerance. Most are stress-responsive genes involved in signaling, transcription, oxidation-reduction process, cell wall and membrane structure, transport, cell cycle and carbohydrate metabolism. Several genes that are strongly up-regulated have not been previously associated with drought or oxidative stress tolerance and will not be discussed here. However, their strong up-regulation suggest that further characterization of these genes may be of interest. Our analysis revealed that in response to drought stress, 188 genes are up-regulated by at least 5-fold in 1B-OEX plants compared to wild-type. It is difficult at this point to determine whether the genes identified in this study are true orthologs of genes whose function in stress tolerance has been demonstrated in transgenic studies. However, they could play similar roles and explain the improvement of drought tolerance in the 1B-OEX plants [72]. A model that summarizes changes in gene expression and their potential relationship to drought tolerance is presented in Fig. 7 to support the discussion.

### Signal perception and modulation

Drought triggers the production of the phytohormone ABA which in turn induces the expression of stress-related genes required for environmental adaptation. ABA-related responses are thus intricately related to drought responses. Leucine-Rich Repeat Receptor-Like Kinases (LRR-RLKs) (#79) belong to the large subfamily of Receptor-Like Kinases (RLKs) which are important mediators of environmental stimuli (Fig. 7). It has been proposed that LRR-RLKs might be involved in early responses to drought and ABA perception [28, 73]. Because of their roles in development and stress responses, LRR-RLKs are new potential targets for abiotic stress tolerance [74]. In Arabidopsis, the repression of the LRR-RLK RPK1 down-regulates many ABA-inducible genes, resulting in a decrease in ABA sensitivity and stomatal closure [28]. This suggests that RLKs function as important regulators in ABA signal transduction pathways. In addition, a LEUNIG and a TOPLESS-related proteins (#41, #87), which are known to act as transcriptional repressors, are induced by drought in 1B-OEX plants [75]. This suggests that transcriptional repression of genes negatively associated with drought tolerance might be a mechanism to study further.

Earlier studies showed that ABA induces ROS production through activation of NADPH oxidases [25]. The increased expression of NADPH oxidase (#143 and Additional file 4: Fig. S3A) in 1B-OEX plants may participate in modulating ABA signaling. ROS such as superoxide radicals and hydrogen peroxide are considered essential molecules in ABA signaling while excessive accumulation can be very toxic during drought stress. Therefore, regulatory mechanisms modulating ROS signal transduction and ROS detoxification are required to orchestrate the responses to ABA. ROS act as intracellular signals to trigger responses to drought stress [76, 77]. They induce phosphorylation and dephosphorylation events through the activation of protein kinases and phosphatases [78]. In this study, several protein kinases and phosphatases up-regulated 2 to 5-fold by overexpression of *TaZFP1B* were identified (Additional file 3: Table S1). Phosphoprotein cascades function as crucial regulators to mediate abiotic stress response and tolerance. Serine/threonine kinases (#81) and MAP kinases (#83) are able to phosphorylate a wide range of substrates and are associated with many different stress responses [79]. Our previous bioinformatic analysis has identified several putative phosphorylation sites for different kinases in the TaZFP1B amino acid sequence [60]. This suggests that phosphorylation may be required to fully activate TaZFP1B under drought stress (Fig. 7). Furthermore, studies have reported that the C2H2 zinc finger proteins ZAT10 and ZAT6 in Arabidopsis, and ZFP36 in rice, require kinase activation for their positive regulation of stress tolerance [57, 80, 81].

To control the level of ROS accumulation under stress, plants activate the expression of genes involved in antioxidant functions and production of stress proteins. In *Arabidopsis*, overexpression of the *ASPG1* aspartic protease resulted in lower H_2_O_2_ levels with the parallel activation of detoxification enzymes (SOD and CAT), enhanced sensitivity to ABA and improved drought tolerance [24]. In accordance, our study revealed that genes encoding aspartic proteases are strongly up-regulated by TaZFP1B in 1B-OEX wheat plants under well-watered conditions (#6) and drought stress (#101, #115 (see also Additional file 4: Fig. S3B), #124). This result indicates that aspartic proteases could play an important role in enhancing drought tolerance and ROS detoxification mechanisms in wheat through the modulation of ABA sensitivity (Fig. 7).

Additionally, ROS accumulation is lower in 1B-OEX plants which is in accordance with the up-regulation of genes encoding SOD, APX, and CAT and the significant increase in SOD, APX and CAT activities. Furthermore, overexpression of *TaZFP1B* increases the expression of a gene encoding an ankyrin repeat containing protein (#25). It was shown that the AKR2A protein acts as a chaperone for APX3 in Arabidopsis [82], providing additional support to the observed increased APX activity. The induction of the Protein DJ-1 homolog D (#86) may contribute to the overall oxidative stress tolerance of 1B-OEX plants since Arabidopsis plants overexpressing AtDJ-1A show increased tolerance against various abiotic stresses, possibly by the interaction with SODs [83]. This suggests that the increased drought tolerance observed in wheat might be also mediated by the interaction with SODs, further linking drought and oxidative stresses. The increase in RPM1 (#88) can also contribute to the oxidative stress improvement, as suggested by the observation that SOD and CAT activities increase when AtRPM1 is overexpressed in Arabidopsis [84]. TaZFP1B OEX also increases GSH and GSSG contents, which are important metabolites for ROS detoxification in plants. It is possible that the increase in cysteine synthase (#90) contributes more Cys for the synthesis of GSH [85]. Together, these results suggest that overexpression of *TaZFP1B* triggers a greater capacity to maintain ROS homeostasis and improves both drought tolerance and productivity compared to wild-type wheat.

An increasing number of studies have reported key roles of ubiquitin-protein ligases (E3s) (#178 and #179 and Additional file 4: Fig. S3C) in plant developmental processes including responses to abiotic stresses [86]. In rice, the U-box E3 ligase OsPUB15 induced by H_2_O_2_ and drought stress plays an important role in plant tolerance. Its overexpression promoted growth under drought stress in transgenic plants [87]. In contrast, overexpression of *AtPUB19* negatively regulated ABA signaling and decreased tolerance to drought stress [88]. Based on these studies, we hypothesize that TaZFP1B function might involve downstream E3 ligases (Fig. 7).

Physical interactions between transcription factors or with other protein complexes have emerged as important mechanisms allowing cross-talk between different pathways that lead to enhanced adaptability to environmental conditions [89]. This suggests that transcription factor members found to be up-regulated in 1B-OEX plants could be involved in specific or shared pathways. As regulators of many stress-responsive genes, transcription factors constitute one of the largest groups of genes differentially expressed in drought-treated 1B-OEX plants. Based on gene annotation, transcription factors belonging to large families are strongly up-regulated: bHLH (#54, #61, #72 and #77), bZIP (#64), CBF/DREB (#49 and #75), ERF (#60), HD-ZIP (#58 and #71), WRKY (#48 and #50) and ZFP (#39, #47, #53, #55, #63, #66, #73 and #76). These transcription factors could induce the expression of additional genes associated with the response to different hormonal signaling pathways involving abscisic acid (#101, #115, #124, #134 and #185), jasmonic acid (JA) (#89 and #107), auxin (#104, #118 and #136), gibberellic acid (GA) (#151), brassinosteroid (BR) (#117) and ethylene (#60) [38]. Many transcription factors within each family are known to enhance drought, salt, cold and osmotic stress tolerance [90, 91] and participate in the regulation of stress responses. Together, these observations suggest that TaZFP1B may be a molecular mediator engaged in a complex network involved in the response to drought stress.

### ROS scavenging and energy supply

Overexpression of *TaZFP1B* resulted in the up-regulation of a number of genes encoding proteins involved in stress tolerance. Overexpression of *TaZFP1B* resulted in lower ROS accumulation and strong induction of ROS scavenging enzymes activity (SOD, APX and CAT). We also found that the NADP-malic enzyme (#183 and Additional file 4: Fig. S3D) is strongly up-regulated in 1B-OEX plants. This enzyme participates in CO_2_ fixation in plants. Additionally, in C3 plants, it is thought to be involved in the conversion of NADH to NADPH which improves cellular antioxidant defense [92]. In tobacco, the NADP-malic enzyme has been associated with drought stress acclimation [93]. Its overexpression resulted in a decrease in stomatal conductance, which improved water use efficiency [94]. This may be useful for the maintenance of growth during drought stress. Other genes involved in ROS scavenging have also been identified in our study. Different classes of glutathione S-transferase (GSTs) up-regulated in 1B-OEX plants (#12, #98 #123, #128, and Additional file 4: Fig. S3E and S3F) are known to promote detoxification of xenobiotics and to participate in the response to various abiotic stresses including oxidative stresses [95, 96]. Some theta, phy and tau GSTs have been shown to have glutathione peroxidase activity to reduce organic hydroperoxides of fatty acids, preventing oxidative damage [97].

Delaying leaf senescence was previously suggested to represent a strategy to enhance drought stress tolerance [98]. Slowing down photosynthesis is one of the main strategies to limit ROS production and propagation through down-regulation of components of the photosynthetic machinery [99]. We found that many genes involved in photosynthesis metabolism are down-regulated in 1B-OEX plants and, contrarily, overexpressed in 1B-siRNA plants during drought stress (Table 5). These genes include RUBISCO activase, RUBISCO small chain, and several other chloroplastic proteins (#244–264). Interestingly, the down-regulation of these genes in 1B-OEX plants is associated with the maintenance of a high chlorophyll content and delayed senescence compared to wild-type and 1B-siRNA plants (Fig. 2 and Additional file 2: Fig. S2). Maintaining the chlorophyll content while uncoupling photosynthesis may help the plant to survive the stress period while allowing it to recover more rapidly after stress.

During water deprivation, energy supply is severely limited. ATP synthase is a key enzyme involved in ATP synthesis during electron transport. Increased expression of ATP synthase (#189) was shown to improve drought tolerance in *Arabidopsis* [100]. Under water shortage, plant energy allocation strategy is crucial to increase survival, therefore the up-regulation of genes encoding bidirectional sugar transporters SWEET (#169, #173 and #174) could contribute to the allocation of energy in proper compartments to support stress tolerance mechanisms [101]. Interestingly, a reduced photosynthesis activity was previously observed in Arabidopsis with a concomitant increase in β-galactosidase activity (#184). The galactosidase activity possibly participates in the catabolic network of cell wall polysaccharides to produce sugars needed as energy source when photosynthate production is lower [102].

### Osmoprotection and structural reinforcement

Osmotic adjustment is one of the most important mechanism used by plants to tolerate drought stress. In our study, we found that genes encoding CBF/DREB (#49, #65 and #75) are up-regulated in 1B-OEX plants under drought stress. The importance of DREB proteins in plant stress signaling and abiotic and biotic stress tolerance was previously reported [103]. Overexpression of CBF/DREB proteins enhances the expression of downstream target genes including Cor14a (#69), dehydrins (#91, #95, #100 and #114) and late embryogenesis abundant proteins (#105, #106) which are known to protect macromolecules from aggregation due to dehydration [104–106].

Additional genes encoding proteins involved in cell structure, elongation and maintenance, and cell wall or membrane metabolism such as xyloglucan endotransglucosylase/hydrolase (#154 and Additional file 4: Fig. S3G), WAX (#158), pectinesterase (#155) and expansin (#156) were also up-regulated by *TaZFP1B* overexpression during drought stress. These proteins play a major role in controlling cell wall extensibility and plasticity, two characteristics required to cope with a progressive decrease in water content. The latter results in considerable mechanical stress on plant cell architecture, therefore increasing elasticity and cellulose synthesis in cell wall would contribute to the maintenance of cell integrity and cell turgor in response to dehydration [17, 107, 108]. Moreover, changes in cell wall polysaccharides and proteins was observed in resurrection plants during drought stress and rehydration [109]. The majority of water loss occurs via transpiration through stomata, but studies have reported that under drought conditions, the synthesis of epicuticular wax on the leaf surface contributes to reducing water loss [110, 111]. WAX (#158) and non-specific lipid transfer protein genes (#172, #175–177 and Additional file 4: Fig. S3H) were reported to promote wax synthesis and to participate in cuticular wax deposition, respectively [110, 112]. Therefore, these results suggest that TaZFP1B regulates stress-responsive genes known for their capacity to enhance adaptation to drought stress (Fig. 7).

### Programmed cell death and growth under stress

Ubiquitins are also involved in the regulation of programmed cell death (PCD) [113]. Abiotic stress-induced PCD is of considerable interest to ensure plant survival since it is a highly regulated process that facilitates the removal of unwanted and damaged cells, thus maintaining tissue and biological process homeostasis. Cell death is an integral part of plant growth and development, and also occurs as part of the plant response to abiotic stresses [114, 115]. In this study, genes regulating PCD and autophagy such as polyamine oxidase (#148), *Laz1* (#160), LOL1 (#164, #165) and LSD1 (#39) were up-regulated in 1B-OEX plants [116–119]. Intriguingly, LOL1 (#164 and #165) and LSD1 (#39) have antagonistic effects on PCD. In *Arabidopsis*, overexpression of *LOL1* enhances pathogen-driven hypersensitive response and oxidative stress-induced cell death [118] while *LSD1* encodes a negative regulator of PCD [119]. Further studies have also reported that LSD1 contributes to plant vegetative and reproductive growth during drought stress by regulating PSII maximum efficiency, water use, cell wall structure and composition, and H_2_O_2_ concentration. This indicates that LSD1 could be involved in the regulation of photosynthesis, transpiration, cell signaling homeostasis, plant biomass production and seed yield [120, 121]. Similarly, emerging evidence suggest that glycine-rich proteins (GRPs) (#113) are involved in the regulation of plant cell growth and drought stress [122]. Overexpression of *Arabidopsis* glycine-rich RNA-binding proteins AtGRP2 or AtGRP7 in rice improves grain yield during drought stress [123], while AtGRP5 promotes cell elongation [124]. These findings suggest that a delicate balance of cell growth and cell death is needed during drought stress acclimation.

## Conclusions

In conclusion, the functional characterization of TaZFP1B at the physiological and molecular levels allowed a better understanding of the importance of this transcription factor in wheat as well as its role in the response and tolerance to drought stress. The use of a novel VOX and VIGS system allowed the rapid functional gene characterization without the need to generate transgenic plant lines, which is a lengthy process in hexaploid wheat. The data presented here indicate that TaZFP1B is a key transcription factor orchestrating multiple molecular mechanisms involving the regulation of a collection of genes associated with stress tolerance, through the activation of multiple signaling pathways or via crosstalks between stress response pathways. The enhanced tolerance to drought or oxidative stress by the overexpression of *TaZFP1B* suggests that this transcription factor could be used as a marker for the selection of drought-tolerant wheat in breeding programs to improve productivity under climate change. Alternatively, a gene editing approach of the promoter region to enhance its expression is an interesting possibility for the improvement of wheat crop productivity under normal growth and drought conditions.

## Methods

### Plant materials and growth conditions

The *Triticum aestivum* cv. Atlas 66 winter wheat cultivar was obtained from Carver and colleagues [125] and was grown in our greenhouse facilities. This cultivar was selected for functional characterization of *TaZFP1B* (GenBank accession number MN577972) since this gene was identified from a previous study in this cultivar and was shown to respond to different abiotic stresses [60, 61]. In addition, to avoid the vernalization step required in winter cultivars, the commercial spring wheat *T. aestivum* cv. Dakosta (https://www.inspection.gc.ca/english/plaveg/pbrpov/cropreport/whe/app00009711e.shtml) was purchased from La Coop Fédérée (Saint-Hyacinthe, Québec Canada) and used to facilitate analysis of drought tolerance during booting. *T. aestivum* and *Nicotiana benthamiana* plants were grown in growth chambers under controlled conditions at 22 °C, a 14 h photoperiod, 100 μmol m^− 2^ s^− 1^ irradiance (fluorescent and incandescent lighting), and 70% relative humidity. Seeds were sown in a mixture of black earth, peat moss and perlite (2:1:1 ratio), and pots were watered every day for 14 days. Drought stress was then initiated by withholding water for different periods, as indicated in the Figure and Table legends. To determine the effect of drought stress on seed yield, water was withheld for 10 days when the developing head within the sheath of the flag leaf became visibly enlarged (booting stage Z45). Control samples were watered daily. Tissue sampling was performed at the same time of day to avoid circadian cycle influence.

### Constructs for *TaZFP1B* silencing and overexpression

The four-component BSMV system developed previously was used for *TaZFP1B* virus-mediated overexpression (VOX) [63]. Compared to the three-component system, this modified system is based on four different plasmids (pCaBS-α, pCaBS-β, pCaBS-γ1 and pCaBS-γ2) where the DNA encoding the γ RNA has been split in two parts to allow for cloning of larger cDNA fragments or to increase expression by cloning genes in the two plasmids: pCaBS-γ1 and pCaBS-γ2. To obtain the *TaZFP1B* coding sequence (447 bp), total RNA was extracted from wheat root tips (cv. Atlas 66) exposed to aluminum, the mRNAs were reverse-transcribed to cDNAs using the SensiFAST™ cDNA Synthesis Kit (Bioline), and the *TaZFP1B* cDNA was PCR-amplified using the Q5 high fidelity DNA polymerase (New England Biolabs). This *TaZFP1B* coding sequence was cloned into pCaBS-γ1 and pCaBS-γ2 vectors using the primer pairs 1B-OEX_LIC1 and 1B-OEX_LIC2 (Additional file 5: Table S2). The resulting plasmids were used with the pCaBS-α and pCaBS-β vectors to maximize *TaZFP1B* overexpression (Fig. 1a) since the expression level is moderate with this system compared to strong promoters generally used in transgenic plants studies.

The three-component BSMV system was used for virus-induced gene silencing (VIGS) of *TaZFP1B* in wheat [126]. This system uses three different plasmids (pCaBS-α, pCaBS-β, pCaBS-γ) that respectively carry DNA sequences encoding the three genomic RNAs (RNAα, RNAβ, RNAγ) of the BSMV strain ND18. The siRNA against *TaZFP1B* was designed using GenScript siRNA Target Finder (https://www.genscript.com/tools/sirna-target-finder). The selected sense-loop-antisense DNA sequence (AAGAGTATTGCGGATCTGAAG**ttgatatccg**CTTCAGATCCGCAATACTCtttttttt) was used to generate the siRNA chimeric construct. To ensure that this siRNA is specific to *TaZFP1B*, BLAST analysis was run against the NCBI and EnsemblPlant databases (release 36) for the wheat genome. The only detectable targets were close members of the same transcription factor family. The siRNA fragment was amplified (see Additional file 5: Table S2 for primers) and subcloned into pCaBS-γ by ligation-independent cloning (LIC). The resulting pCaBS-γ:1B-siRNA vector was used with pCaBS-α and pCaBS-β for the silencing of *TaZFP1B* in wheat (Fig. 1b).

### Agroinfiltration of *Nicotiana benthamiana* and viral inoculation in wheat

Viral extracts to be used either for overexpression or silencing of *TaZFP1B* in wheat were produced in *N*. *benthamiana*. The plasmids (pCaBS-α, pCaBS-β, pCaBS-γ:*TaZFP1B*-siRNA for silencing; pCaBS-α, pCaBS-β, pCaBS-γ1:*TaZFP1B* and pCaBS-γ2:*TaZFP1B* for overexpression), were transformed individually into *Agrobacterium tumefaciens* strain EHA105 by electroporation. Single colonies were grown overnight at 28 °C in LB containing rifampicin (25 μg/ml) and kanamycin (50 μg/ml). The cultures were diluted 1:100 with LB containing the same antibiotics, 10 mM 2-(N-morpholino) ethanesulfonic acid (MES) pH 5.2 and 20 μM acetosyringone, and grown at 28 °C for 12 h. Bacterial cells were pelleted at 2200 g and equal amounts of the three or four *Agrobacterium* cultures (OD_600_ = 0.700) were mixed and incubated for 3–5 h at 28 °C in infiltration buffer (10 mM MES, pH 5.2, 10 mM MgCl_2_ and 0.1 mM acetosyringone) as described previously [126]. Agroinfiltration of *N. benthamiana* leaves and preparation of the homogenates containing the viral extracts were done as described previously [63, 127]. These extracts were aliquoted in small volumes and stored at − 20 °C for later use. As control for BSMV infection, empty vectors (pCaBS-γ1:00 and pCaBS-γ2:00) were used with the pCaBS-α, pCaBS-β vectors. Wheat seeds were inoculated with viral extracts for 3 days during seed imbibition, as previously described [63, 127]. Infected seeds were sown in potting mixture at a density of 10 per pot.

Four different types of plants were used in this study: wild-type, plants transformed with the BSMV vectors not containing the *TaZFP1B* cDNA (empty-vector), plants overexpressing *TaZFP1B* (1B-OEX) and plants under expressing *TaZFP1B* (1B-siRNA).

### Determination of growth parameters

Plants were harvested at different times as described for each experiment. Entire plants (roots and shoots) separately were weighed before and after drying at 70 °C for 3 days, and the following growth parameters were assessed: dry weight, plant height, shoot-root ratio calculated from dry weight, second leaf width, and relative water content (RWC). RWC was measured as described previously [128]. Survival rates were calculated from wheat plants that were able to regrow after water withholding and rewatering. The effect of drought stress on grain yield (number and weight) was assessed in the spring wheat cultivar Dakosta by applying drought stress during the booting stage as described above.

### Imaging of chlorophyll luminescence

Chlorophyll luminescence was imaged using an in vivo imaging system that includes a light-tight cabinet and a charged-coupled-device (CCD) camera (NightOWL II LB983, Berthold Technologies) with an exposure time of 0.6 s. Image analysis was performed with the IndiGO software (Berthold).

### RNA isolation, RNA-Seq library preparation, sequencing and analyses

Total RNA samples of wild-type and BSMV-infected plants (cv. Atlas 66) were harvested from the second leaf of three 21 day-old well-watered wheat plants (control) and three 14 day-old plants that were drought-stressed for 7 days (total of 21 days of growth). The tissue was flash-frozen in liquid nitrogen and total RNA was extracted with the RNeasy Plant Mini kit (QIAGEN) according to the manufacturer’s instructions. RNA samples were treated with on-column RNase-free DNase (QIAGEN) to remove any contaminating genomic DNA. The quality of RNA samples with OD_260_/OD_280_ values greater than 2.0 was assessed by electrophoresis. Determination of RNA Integrity Number (RIN) value, preparation and sequencing of RNA libraries were performed at Novogene (California, USA). Paired-end (2 × 150 b) sequencing was performed on RNA-Seq libraries on the Illumina NovaSeq platform, and we analysed the raw data provided by Novogene. Each read of the paired-end sequences was analysed separately and the transcript per million values were then compared to exclude inconsistent results (more than five-fold difference). The quality of the raw data was verified with FastQC [129]. Adapters (TruSeq3 pair ended) were trimmed using Trimmomatic (version 0.36.3) [130]. Reads were globally filtered to retain reads with base quality exceeding Q30. The RNA-Seq data were cleaned to remove adapters, low read quality and artifact sequences. Transcriptome analysis was performed using the Galaxy pipeline https://usegalaxy.org [131]. Sequence artifacts were removed using a FASTX-toolkit available on the Galaxy platform. The similarity between RNA-Seq datasets was evaluated using plotCorrelation [132]. A heat map plotting type based on the Spearman correlation method was generated to visualize correlations between the libraries and reads from both ends of the paired-end sequencing. To quantify the expression of transcripts, Salmon (version 0.8.2) was used in quasi-mapping-mode [133]. Kmer size was set at 31. Wheat transcriptomic data (Database version 90.3) was retrieved from Ensembl Plants (ftp://ftp.ensemblgenomes.org/pub/plants/release-37/fasta/triticum_aestivum/cdna/) and selected as reference transcriptome for transcript quantification [134]. The transcript per million values of the transcripts generated by Salmon on the RNA-Seq data was used to calculate the splicing ratio. Genes with zero or low counts were excluded by applying a feature selection (Additional file 3: Table S1 and Additional file 6: Table S3). For analysis, we selected genes that are overexpressed 5-fold or more in 1B-OEX plants compared to wild-type plants either under well-watered conditions or under drought stress. Genes that showed similar expression in the empty vector and 1B-OEX RNA-Seq data were excluded. We also selected a few genes of particular interest such as ROS detoxifying enzymes that were overexpressed less than 5-fold in the RNA-Seq data. In this case, their expression level was quantified by qRT-PCR. The heatmapper program (http://www.heatmapper.ca/expression/) was used to draw the heatmap of the expression data. Annotation of transcripts was done based on the wheat annotated genomic transcripts (version release 37) available on Ensembl Plants (ftp://ftp.ensemblgenomes.org/pub/plants/release-37/fasta/triticum_aestivum/) or by BLAST search against the NCBI nucleotide collection (nr/nt). De novo transcriptome assemblies were performed using Trinity [135] to determine the real sequences of genes in wheat cv. Atlas 66. The RNA-Seq data were deposited at NCBI Gene Expression Omnibus (GEO) under the accession number GSE136683.

### Quantitative RT-PCR analysis

Total RNA used for quantitative real-time polymerase chain reaction (qRT-PCR) analysis was extracted from the second leaf of wheat (cv. Atlas 66) plants as described above. Total RNA (0.5 μg) was reverse-transcribed in a total volume of 20 μl using the SensiFAST™ cDNA Synthesis Kit (Bioline). Real-time quantitative RT-PCR was performed on a CFX96 Touch™ Thermal Cycler (Bio-Rad) using the Luna® Universal qPCR Master Mix (New England Biolabs). To quantify specific transcripts from homoeologous genes, primers in Additional file 5: Table S2 were designed based on sequences retrieved from RNA-Seq data of cv. Atlas 66. For each gene, the reference gene sequence was retrieved from Ensembl Plants database and aligned to the assembled transcriptome generated by Trinity. The sequence of interest was identified based on the lowest E-value. Amplification was performed as follows: 5 min at 95 °C followed by 35 cycles of 95 °C for 15 s, 58 °C for 30 s. The target gene transcript level was normalized against the 18S rRNA level. Fold changes of RNA transcripts were calculated using the 2^-ΔΔCt^ method [136].

### ROS detection

Each leaf sample (0.1 g) was ground in liquid nitrogen and the powder was mixed in a microtube with 1 mL of 10 mM Tris-HCl, pH 7.2, then centrifuged at 12,000 *g* for 20 min at 4 °C. The supernatant was transferred to a fresh microtube and used for total ROS and H_2_O_2_ detection. Detection of total ROS was performed using the fluorogenic dye 2′,7′-dichlorodihydrofluorescein diacetate (DCFDA), which is deacetylated by cellular esterases to a compound that can be oxidized by ROS to 2′,7′-dichlorofluorescein (DCF) [137]. DCF fluorescence was measured using a microplate reader with excitation and emission wavelengths of 495 nm and 529 nm, respectively. The non-specific background was subtracted from experimental values by performing other measurements after treatment with catalase (300 units/mL) at room temperature for 10 min. The corrected fluorescence values were expressed as relative fluorescence units/mg of protein extract. Detection of hydrogen peroxide (H_2_O_2_) was carried out using the Hydrogen Peroxide Fluorescent Detection Kit (Arbor Assays, USA), using excitation and emission wavelengths of 570 nm and 590 nm, respectively. H_2_O_2_ concentration was calculated from a standard curve and expressed as μmol/g of fresh weight. The DCF and H_2_O_2_ data were normalized to corresponding well-watered wild-type plants.

### Lipid peroxidation detection

Oxidative damage to lipids in leaf tissue was estimated using the thiobarbituric acid reactive substances (TBARS) test which determines the content of malondialdehyde (MDA). Each leaf sample (0.5 g) was ground in a mortar with 0.5% (w/v) trichloroacetic acid (TCA) and the homogenate was centrifuged at 12,000 *g* for 20 min. The supernatant was transferred to a glass tube and mixed with a solution containing 40% TCA and 1% (w/v) thiobarbituric acid (TBA). The mixture was incubated in boiling water for 30 min and the reaction was stopped by moving the tube to an ice bath. The mixture was then mixed with 1-butanol and vortexed vigorously for 10 s, then phases were separated by centrifugation (4000 *g*, 5 min at room temperature). The upper phase was carefully transferred to a new tube and absorbance values were read at 532 nm and 600 nm. The latter value represents non-specific absorption and was subtracted. The content of MDA-TBA complex in the samples was calculated as MDA equivalent from an MDA standard curve (Cayman Chemical, USA). The MDA data was normalized to corresponding well-watered wild-type plants.

### Determination of antioxidant components: enzymes and metabolites

Total activity of superoxide dismutase (SOD; EC 1.15.1.1) was assayed by the inhibition of photochemical reduction of nitroblue tetrazolium (NBT) as described previously [138]. Each leaf sample (0.5 g) was ground with liquid nitrogen and suspended in 1.5 mL of homogenization buffer: 50 mM sodium phosphate (pH 7.8), 1 mM ethylenediaminetetraacetic acid (EDTA) and 2% (w/v) polyvinylpolypyrrolidone (PVPP). The homogenate was centrifuged at 12,000 *g* for 30 min at 4 °C and the supernatant was immediately used for the SOD assay. The 3.0 mL assay medium contained 50 mM sodium phosphate (pH 7.8), 0.66 mM EDTA, 10 mM L-methionine, 33 μM nitroblue tetrazolium (NBT) and 3.3 μM riboflavin. The reaction was started by placing the tubes under light (600 μmol m^− 2^ s^− 1^) for 10 min at 25 °C. Reduction of NBT was measured at 560 nm. One enzyme unit of SOD is defined as the amount of protein causing a 50% inhibition of NBT photoreduction. The results were expressed as units/mg of soluble proteins.

Total ascorbate peroxidase activity (APX; EC 1.11.1.11) was determined by following the decrease in A_290_ for 2 min at 25 °C [139]. Each leaf sample (0.5 g) was ground with liquid nitrogen and suspended in 1.5 mL of homogenization buffer containing 50 mM sodium phosphate (pH 7.0), 2% (w/v) PVPP, 0.1 mM EDTA and 2 mM ascorbate. The homogenate was centrifuged at 12,000 *g* for 30 min at 4 °C. The reaction mixture (1 mL) contained 50 mM sodium phosphate, 0.1 mM EDTA, 0.5 mM ascorbate and 0.5 mM H_2_O_2_ and the reaction rate (ascorbate oxidized/min) was measured at 22 °C. The molar extinction coefficient of ascorbate (ε) used for calculation was 2.8 mM^− 1^ cm^− 1^. The results were expressed as units of APX (μmol ascorbate) per mg of soluble proteins.

Catalase activity (CAT; EC 1.11.1.6) was determined by following the consumption of H_2_O_2_ at 240 nm for 2 min at 25 °C [140]. Each leaf sample (0.5 g) was ground with liquid nitrogen and suspended in 1.5 mL of homogenization buffer containing 50 mM Tris-HCl (pH 7.8), 0.1 mM EDTA, 0.2% (v/v) Triton X-100, 1 mM phenylmethylsulphonyl fluoride (PMSF) and 2 mM dithiothreitol (DTT). The homogenate was centrifuged at 12,000 *g* for 30 min at 4 °C and the supernatant was used for the CAT assay. The reaction mixture contained 50 mM sodium phosphate (pH 7.0) and 0.1% H_2_O_2_ and the reaction rate (μmol H_2_O_2_ consumed/min) was measured at 22 °C. The molar extinction coefficient of H_2_O_2_ (ε) used for calculation was 39.4 mM^− 1^ cm^− 1^. The results were calculated as units of CAT (μmol H_2_O_2_) per mg soluble proteins.

For glutathione determination, each leaf tissue (0.1 g) was ground in liquid nitrogen and the powder was placed in a microtube with 1 mL of 10 mM Tris-HCl, pH 7.2 and centrifuged at 12,000 *g* for 20 min at 4 °C. The supernatant was transferred to a fresh microtube. Reduced glutathione (GSH) and oxidized glutathione (GSSG) levels were determined using the Glutathione (GSH) Fluorescent Detection Kit (Arbor Assays, USA) according to the manufacturer’s protocol. A standard curve of GSH was established to determine the concentrations of free GSH and total GSH in samples. The results were expressed as μmol per mg soluble proteins.

### Protein quantification

Proteins were quantified using the Bio-Rad Protein Assay Dye Reagent (Bio-Rad, Canada). Absorbance was read at 595 nm with a microplate reader, and protein concentration was determined using a standard curve prepared with bovine serum albumin (BSA).

### Statistical analyses and validation of RNA-Seq data

All experiments with the exception of RNA-Seq were repeated at least four times independently and the value presented are means ± standard deviations (SD). Comparisons of means were conducted using one-way analysis of variance (ANOVA). Differences among means were analyzed using Tukey’s post hoc test at *p* values < 0.05. Statistical analyses were performed using InStat 3.0. Pairwise correlation was performed to analyze the relationship between libraries, and a heat map was generated to verify the correlation between both ends of paired end sequencing of each treatment (Additional file 7: Fig. S4). The heatmap reveals a close relationship between the wild-type and the empty vector treatment, indicating that there is no major change in gene expression due to the BSMV infection itself. The analysis shows that the well-watered and drought libraries have a low correlation, indicating profound changes in wheat transcriptome profiles when drought stress is applied. Additionally, the correlation between the 1B-OEX and 1B-siRNA libraries is the lowest whether comparing well-watered or drought-treated plants, indicating significant differences in gene expression profiles. This suggests that several genes that are regulated by the expression of *TaZFP1B* are likely regulated in an opposite manner in the 1B-siRNA plants. For the qRT-PCR validation of RNA-Seq results, 16 genes up-regulated in drought-stressed 1B-OEX were randomly selected from the lists of differentially regulated transcripts in the RNA-Seq data and expression levels were analysed using specific primers between drought-stressed 1B-OEX and wild-type plants (Additional file 5: Table S2). Scatterplots were generated by comparing the log_2_ fold change (OEX drought/wild-type drought). The results show that the correlation between the expression patterns obtained by qPCR and the RNA-Seq data (*R*^2^ = 0.6374, *p* > 0.01) (Additional file 8: Fig. S5) is similar to what is observed between qPCR and microarray data.

## Supplementary information

**Additional file 1: Figure S1.** Silencing of *TaZFP1B* affects relative expression of the closest *TaZFP1B* relatives. The different types of wheat plants (see Fig. 1) were grown for 14 days then were either well-watered for an additional 7 days or drought-stressed by withholding water for 7 days, and expression levels were determined by qRT-PCR. Data are the mean expression ± SD of four biological replicates. Different letters indicate statistically significant differences between samples (*P* < 0.05 by Tukey’s test).

**Additional file 2: Figure S2.** Chlorophyll autofluorescence from wheat leaves. The different types of wheat plants (see Fig. 1) were grown for 14 days then were either well-watered for an additional 10 or 14 days (top leaf in the panels) or drought-stressed by withholding water for 10 or 14 days (bottom leaf in the panels). Fluorescence was captured using a NightOWL II imaging cabinet.

**Additional file 3: Table S1.** List of genes up-regulated by TaZFP1B overexpression.

**Additional file 4: Figure S3.** Validation of RNA-Seq data by qRT-PCR. The different types of wheat plants (see Fig. 1) were grown for 14 days then were either well-watered for an additional 7 days or drought-stressed by withholding water for 7 days. Expression levels are relative to the well-watered wild-type group. Numbers refer to the corresponding genes in Tables 2, 3, 4 and 5. Data are mean expression ± SD of four biological replicates. Different letters indicate statistically significant differences between samples (*P* < 0.05 by Tukey’s test).

**Additional file 5: Table S2.** List of genes down-regulated by TaZFP1B overexpression.

**Additional file 6: Table S3.** Primers used in this study.

**Additional file 7: Figure S4** Heatmap of correlations between RNA-seq libraries. The different types of wheat plants (see Fig. 1) were grown for 14 days then were either well-watered for an additional 7 days or drought-stressed by withholding water for 7 days. RNA-Seq libraries were prepared and paired-end sequencing was performed. Each read generated from paired-end sequencing was analyzed individually. The hierarchical clustering was generated using Spearman correlation coefficient. The color scale indicates the degree of correlation.

**Additional file 8: Figure S5.** Pearson correlation between the RNA-seq and qRT-PCR data. The qRT-PCR log2 value of the expression ratio (drought-treated wild-type/drought-treated 1B-OEX) (y-axis) was plotted from the RNA-seq log2 value of expression ratio (drought-treated wild-type/drought-treated 1B-OEX) (x-axis). Genes used to calculate the correlation are listed in supplementary Table 1. All qRT-PCR data were collected from three biological replicates. --- represents the 95% confidence interval. The calculated correlation value (*R*^2^) is shown along with the regression line.

## Data Availability

The RNA-Seq datasets generated and/or analysed during the current study are available in the GEO repository under the accession number GSE136683, https://www.ncbi.nlm.nih.gov/geo/query/acc.cgi?acc=GSE136683. The *TaZFP1B* sequence is available at GenBank under accession number MN577972 (https://www.ncbi.nlm.nih.gov/nuccore/MN577972).

## Abbreviations

ABA: Abscisic acid
APX: Ascorbate peroxidase
ATP: Adenosine triphosphate
bHLH: basic helix-loop-helix protein
BSA: Bovine serum albumin
BSMV: Barley stripe mosaic virus
bZIP: basic leucine zipper
CaM1: Calmodulin 1
CAT: Catalase
CBF: C-repeat binding factors
DCF: 2′,7′-dichlorofluorescein
DREB: Drought response element binding protein
DTT: Dithiothreitol
EDTA: Ethylenediaminetetraacetic acid
ERF: Ethylene responsive factor
GRP: Glycine-rich proteins
GSH: Reduced glutathione
GSSG: Oxidized glutathione
GST: Glutathione S-transferase
H_2_O_2_: Hydrogen peroxide
HD-ZIP: Homeodomain-leucine zipper transcription factor
JA: Jasmonic acid
Laz 1: Lazarus 1
LEA: Late embryogenesis abundant
LOL1: Lsd one like 1
LRR-RLK: Leucine-rich repeat receptor-like kinases
MDA: Malondialdehyde
MYB: Myeloblastosis oncogene
NAC: NAM, ATAF1/2 and CUC
NADPH: Reduced nicotinamide adenine dinucleotide phosphate
NBT: Nitroblue tetrazolium
NF-Y: Nuclear factor Y
PCD: Programmed cell death
PMSF: Phenylmethylsulphonyl fluoride
PVPP: Polyvinylpolypyrrolidone
ROS: Reactive oxygen species
RPK 1: Receptor-like kinase 1
RUBISCO: Ribulose-1,5-bisphosphate carboxylase/oxygenase
RWC: Relative water content
SLAC: Slow anion channel
SOD: Superoxide dismutase
SWEET: Sugars will eventually be exported transporters
TBA: Thiobarbituric acid
TBARS: Thiobarbituric acid reactive substances
TCA: Trichloroacetic acid
VIGS: Virus-induced gene silencing
VOX: Virus-mediated overexpression
WRKY: Proteins containing the highly conserved amino acid sequence WRKYGQK
XTH: Xyloglucan endotransglucosylase/hydrolase
ZFP: Zinc finger proteins
