# Supplementary figures and images for: The barley stripe mosaic virus expression system reveals the wheat C2H2 zinc finger protein TaZFP1B as a key regulator of drought tolerance
Source: BMC Plant Biol. 2020 Apr 7;20:144. doi: 10.1186/s12870-020-02355-x (PMC7140352; doi:10.1186/s12870-020-02355-x)

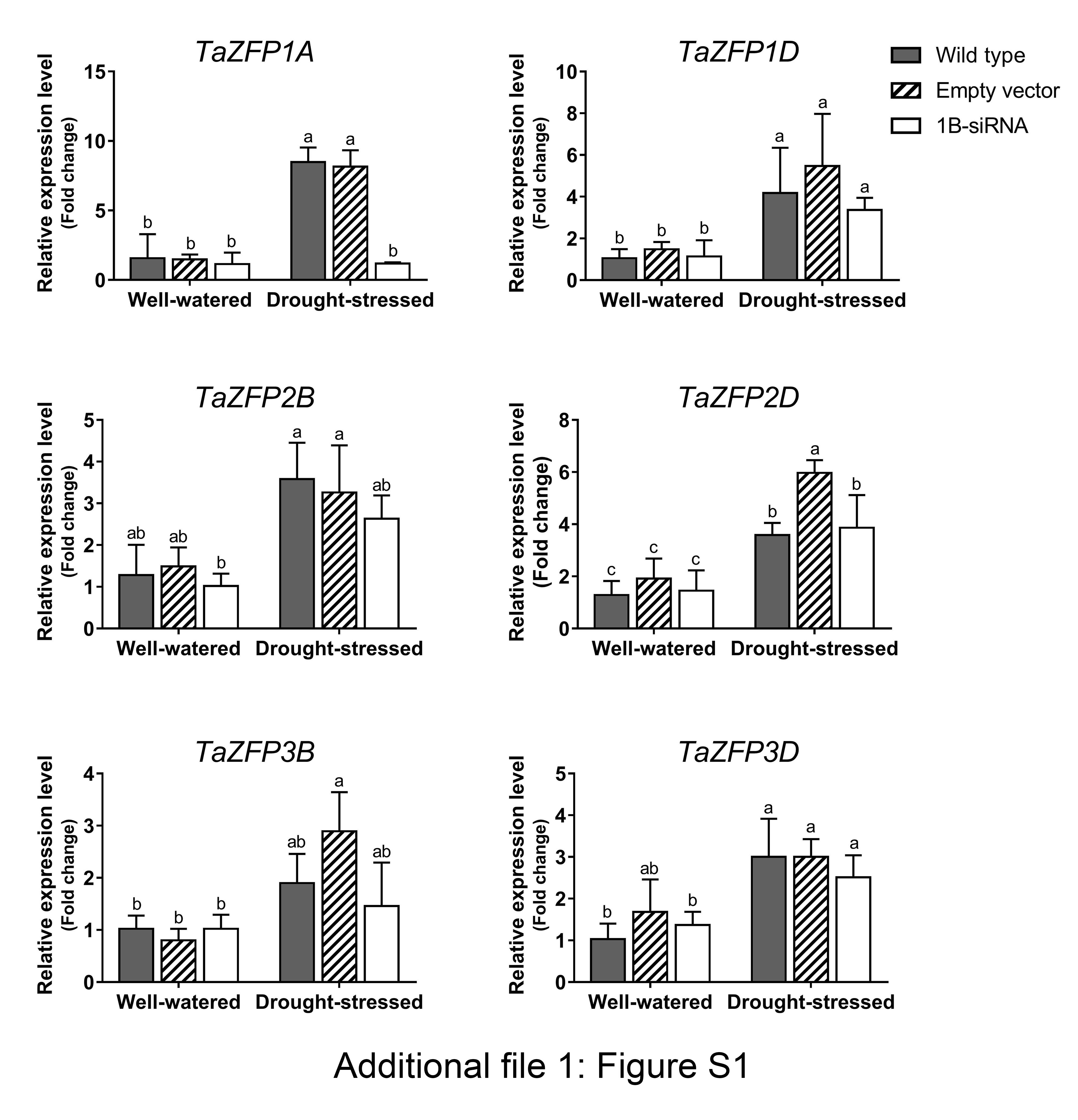

Supplement: Supplementary file 1 — Additional file 1: Figure S1. Silencing of TaZFP1B affects relative expression of the closest TaZFP1B relatives. The different types of wheat plants (see Fig. 1) were grown for 14 days then were either well-watered for an additional 7 days or drought-stressed by withholding water for 7 days, and expression levels were determined by qRT-PCR. Data are the mean expression ± SD of four biological replicates. Different letters indicate statistically significant differences between samples (P < 0.05 by Tukey’s test). [file 12870_2020_2355_MOESM1_ESM.jpg]

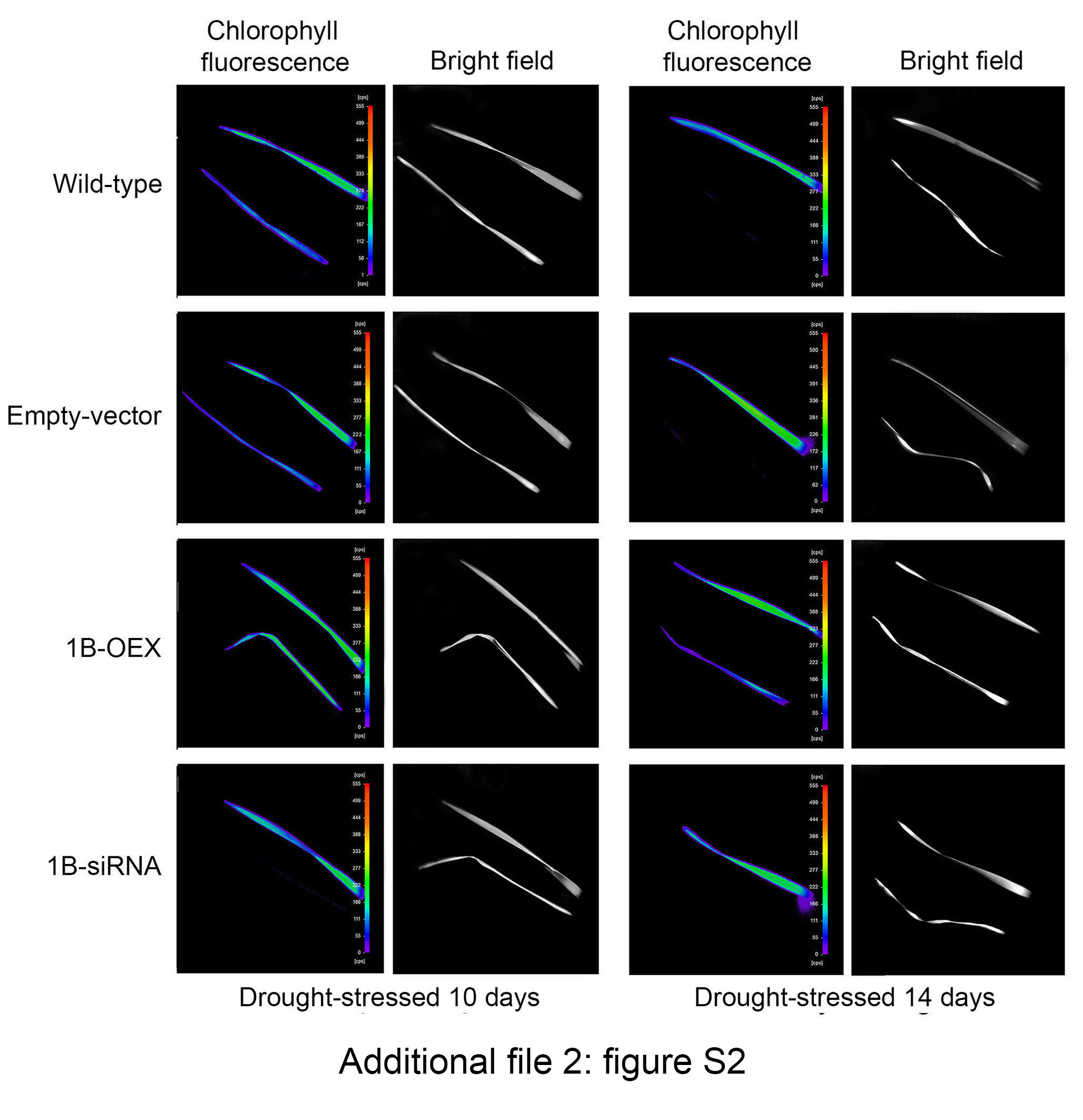

Supplement: Supplementary file 2 — Additional file 2: Figure S2. Chlorophyll autofluorescence from wheat leaves. The different types of wheat plants (see Fig. 1) were grown for 14 days then were either well-watered for an additional 10 or 14 days (top leaf in the panels) or drought-stressed by withholding water for 10 or 14 days (bottom leaf in the panels). Fluorescence was captured using a NightOWL II imaging cabinet. [file 12870_2020_2355_MOESM2_ESM.jpg]

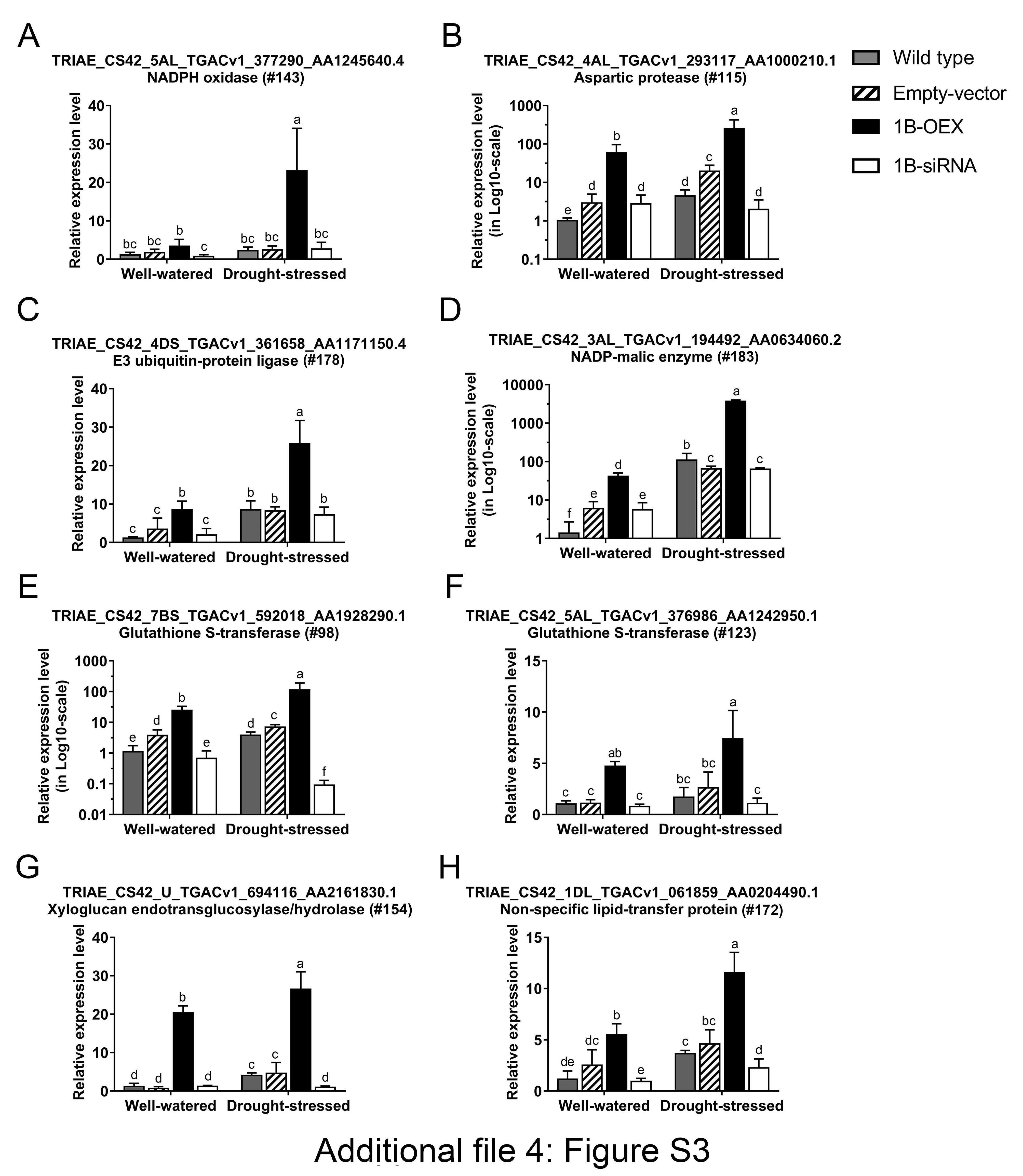

Supplement: Supplementary file 4 — Additional file 4: Figure S3. Validation of RNA-Seq data by qRT-PCR. The different types of wheat plants (see Fig. 1) were grown for 14 days then were either well-watered for an additional 7 days or drought-stressed by withholding water for 7 days. Expression levels are relative to the well-watered wild-type group. Numbers refer to the corresponding genes in Tables 2, 3, 4 and 5. Data are mean expression ± SD of four biological replicates. Different letters indicate statistically significant differences between samples (P < 0.05 by Tukey’s test). [file 12870_2020_2355_MOESM4_ESM.jpg]

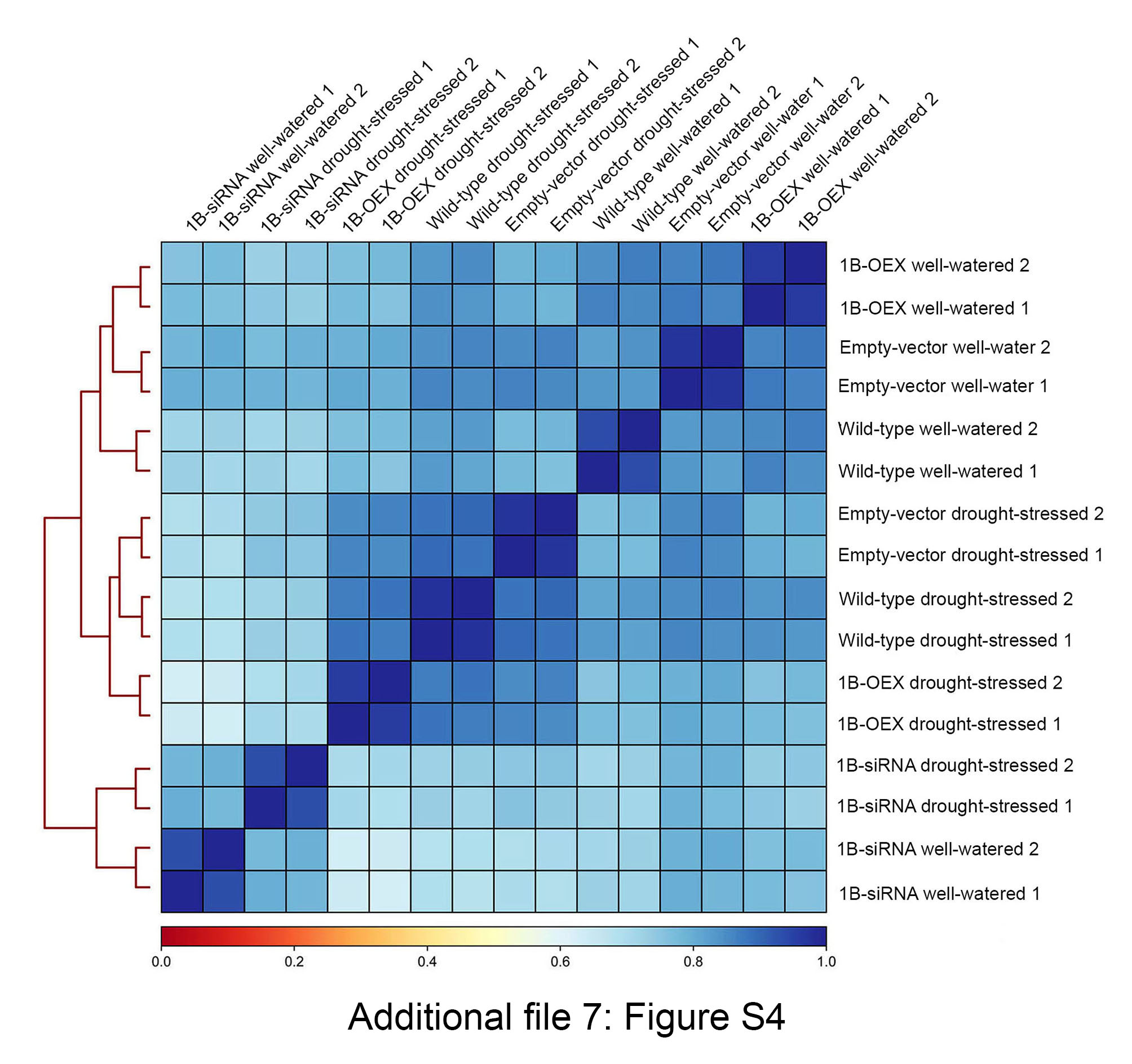

Supplement: Supplementary file 7 — Additional file 7: Figure S4 Heatmap of correlations between RNA-seq libraries. The different types of wheat plants (see Fig. 1) were grown for 14 days then were either well-watered for an additional 7 days or drought-stressed by withholding water for 7 days. RNA-Seq libraries were prepared and paired-end sequencing was performed. Each read generated from paired-end sequencing was analyzed individually. The hierarchical clustering was generated using Spearman correlation coefficient. The color scale indicates the degree of correlation. [file 12870_2020_2355_MOESM7_ESM.jpg]

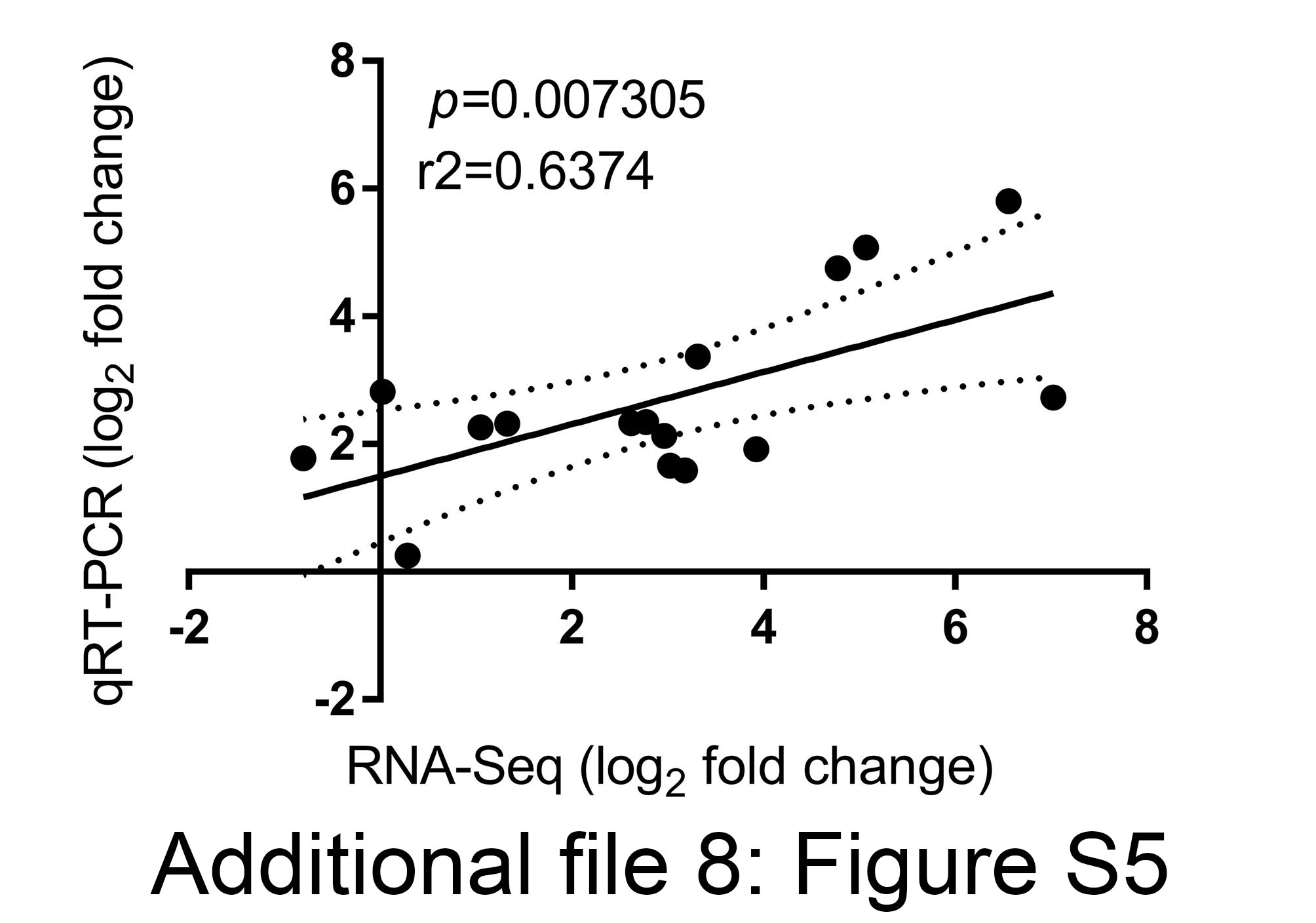

Supplement: Supplementary file 8 — Additional file 8: Figure S5. Pearson correlation between the RNA-seq and qRT-PCR data. The qRT-PCR log2 value of the expression ratio (drought-treated wild-type/drought-treated 1B-OEX) (y-axis) was plotted from the RNA-seq log2 value of expression ratio (drought-treated wild-type/drought-treated 1B-OEX) (x-axis). Genes used to calculate the correlation are listed in supplementary Table 1. All qRT-PCR data were collected from three biological replicates. --- represents the 95% confidence interval. The calculated correlation value (R2) is shown along with the regression line. [file 12870_2020_2355_MOESM8_ESM.jpg]
