# Supplementary material for: The barley stripe mosaic virus expression system reveals the wheat C2H2 zinc finger protein TaZFP1B as a key regulator of drought tolerance
Source: BMC Plant Biol. 2020 Apr 7;20:144. doi: 10.1186/s12870-020-02355-x (PMC7140352; doi:10.1186/s12870-020-02355-x)
Supplement: Supplementary file 6 — Additional file 6: Table S3. Primers used in this study. [file 12870_2020_2355_MOESM6_ESM.docx]

Additional file 6: Table S3 Primers used in this study

| Name | Forward primer | Reverse primer | Usage |
| --- | --- | --- | --- |
| 1B-siRNA_LIC | AAGGAAGTTTAAAAGAGTATTGCGGATCTGAAGTTGA | AACCACCACCACCGTAAAAAAAAGAGTATTGCGGATCTGA | Cloning of the 1B-siRNA fragment into pCaBS-γ (γ:TaZFP1B-siRNA) |
| 1B-OEX_LIC1 | AAGGAAGTTTAAATGACCAAGCACCAGAGAGC | AACCACCACCACCGTAGTGCAGATGCAGAGTTTCC | Cloning of the *TaZFP1B* coding sequence into pCaBS-γ1 (γ1:TaZFP1B-OEX) |
| 1B-OEX_LIC2 | AAGGAAGTTTAAATGACCAAGCACCAGAGAGC | CGGGCCAGCCACCGCCACCAGTAGTGCAGATGCAGAGTTTCC | Cloning of the*TaZFP1B* coding sequence into pCaBS-γ2 (γ2:TaZFP1B-OEX) |
| 1B_qRT-PCR | CACCGGACTAGCCACCTG | CCTGCTTCAGATCCGCAAT | qRT-PCR of TaZFP1B (TRIAE_CS42_5BL_TGACv1_409557_AA1366220.1) |
| NADPHox | CAAAGGCTTGCAGTTTGTGA | CCCAAATCTCGAACGGAGTA | qRT-PCR of NADPH oxidase (TRIAE_CS42_5AL_TGACv1_377290_AA1245640.4) |
| GST7B | GATGATGCGCTACTTCATGG | TCTCTGCCTTCTTTCCAAGC | qRT-PCR of glutathione S-transferase (TRIAE_CS42_7BS_TGACv1_592018_AA1928290.1) |
| GST5A | CCCAACACTGATCTCCTCGT | CTTTAGAGGGCCGTGGTACA | qRT-PCR of glutathione S-transferase (TRIAE_CS42_5AL_TGACv1_376986_AA1242950.1) |
| SOD | GAGGGTGCTGCTTTACAAGG | CCAACAAAGGATGCAGGTTT | qRT-PCR. of superoxide dismutase (TRIAE_CS42_2BL_TGACv1_131439_AA0427700) |
| APX | TGGCATGACGCTGGTACTTA | CACTGGCAAGCTGAAACAGA | qRT-PCR. of ascorbate peroxidase (TRIAE_CS42_U_TGACv1_642188_AA2112960.5) |
| CAT | TCAAGCCAAGTCCAAAGACC | TCCATCCCTGCTGATTAAGG | qRT-PCR of catalase (TRIAE_CS42_7DL_TGACv1_602975_AA1973160.1) |
| ASPG4A | GAGGACACCTTCACCTTCCA | CATGGGGCTGGTTTTGTACT | qRT-PCR of aspartic protease  (TRIAE_CS42_4AL_TGACv1_293117_AA1000210.1) |
| ADH2H | TCGACAAAGTCTGCCTCCTT | CAGCTCCAAGACCGAAAATC | qRT-PCR of alcohol dehydrogenase ADH2H (TRIAE_CS42_5AL_TGACv1_376972_AA1242830.1) |
| NADP-ME | TGCCGTGAAGCAGAACTATG | CACCAGCAAACTTCAGACCA | qRT-PCR of NADP-dependent malic enzyme (TRIAE_CS42_3AL_TGACv1_194492_AA0634060.2) |
| XTH | ACCCCAAGCACATCATCTTC | GGCTGGTTCTTGGGGAAT | qRT-PCR of xyloglucan endotransglucosylase/hydrolase (TRIAE_CS42_U_TGACv1_694116_AA2161830.1) |
| nsLTP1D | GCTTCATGGTTGTGGCTCTC | TGTACTTGCACAGGCAGCTC | qRT-PCR of a putative non-specific lipid-transfer protein (TRIAE_CS42_1DL_TGACv1_061859_AA0204490.1) |
| eif5A | CCACTACCTATCATCCGTGACA | CGTGCTTTCTGGCATACAAT | qRT-PCR of eukaryotic translation initiation factor 5A (TRIAE_CS42_2AS_TGACv1_112557_AA0340950.6) |
| eif5B | AAGGGTGAAAGCAATCATGG | ATCTGGTATGGCAGGGTTTG | qRT-PCR of Eukaryotic translation initiation factor 5B (TRIAE_CS42_5BS_TGACv1_423537_AA1378900.2) |
| Ubi | CGCAGTCTCTTCATTCACCA | CACACGTTGCATTGGACACT | qRT-PCR of E3 ubiquitin-protein ligase (TRIAE_CS42_4DS_TGACv1_361658_AA1171150.4) |
| COR14a | GTGGAGGGAGCCAAGGAC | CATTTGCTCACATCCTCGAC | qRT-PCR of cold-responsive protein COR14a (TRIAE_CS42_2DL_TGACv1_162669_AA0563320.1) |

In the primer sequences, LIC sequences/adaptors are underlined. The sequence of gene accession number (in parenthesis) are available on EnsemblPlants (*Triticum* *aestivum*, release 37).
